# Benchmarking Density Functional Approximations for Excited-State Properties of Fluorescent Dyes

**DOI:** 10.3390/molecules26247434

**Published:** 2021-12-08

**Authors:** Anna M. Grabarz, Borys Ośmiałowski

**Affiliations:** 1 Faculty of Chemistry, Wrocław University of Science and Technology, Wyb. Wyspiańskiego 27, PL-50370 Wrocław, Poland; 2 Faculty of Chemistry, Nicolaus Copernicus University, Gagarina 7, PL-87100 Toruń, Poland; borys.osmialowski@umk.pl

**Keywords:** TD-DFT, ab initio, fluorescent dyes, vertical energy, dipole moments

## Abstract

This study presents an extensive analysis of the predictive power of time-dependent density functional theory in determining the excited-state properties of two groups of important fluorescent dyes, difluoroboranes and hydroxyphenylimidazo[1,2-a]pyridine derivatives. To ensure statistically meaningful results, the data set is comprised of 85 molecules manifesting diverse photophysical properties. The vertical excitation energies and dipole moments (in the electronic ground and excited states) of the aforementioned dyes were determined using the RI-CC2 method (reference) and with 18 density functional approximations (DFA). The set encompasses DFAs with varying amounts of exact exchange energy (EEX): from 0% (e.g., SVWN, BLYP), through a medium (e.g., TPSSh, B3LYP), up to a major contribution of EEX (e.g., BMK, MN15). It also includes range-separated hybrids (CAM-B3LYP, LC-BLYP). Similar error profiles of vertical energy were obtained for both dye groups, although the errors related to hydroxyphenylimidazopiridines are significantly larger. Overall, functionals including 40–55% of EEX (SOGGA11-X, BMK, M06-2X) ensure satisfactory agreement with the reference vertical excitation energies obtained using the RI-CC2 method; however, MN15 significantly outperforms them, providing a mean absolute error of merely 0.04 eV together with a very high correlation coefficient ($R^{2}$ = 0.98). Within the investigated set of functionals, there is no single functional that would equally accurately determine ground- and excited-state dipole moments of difluoroboranes and hydroxyphenylimidazopiridine derivatives. Depending on the chosen set of dyes, the most accurate $\mu_{\mathrm{GS}}$ predictions were delivered by MN15 incorporating a major EEX contribution (difluoroboranes) and by PBE0 containing a minor EEX fraction (hydroxyphenylimidazopiridines). Reverse trends are observed for $\mu_{\mathrm{ES}}$, i.e., for difluoroboranes the best results were obtained with functionals including a minor fraction of EEX, specifically PBE0, while in the case of hydroxyphenylimidazopiridines, much more accurate predictions were provided by functionals incorporating a major EEX contribution (BMK, MN15).

## 1. Introduction

Unceasing development of various technologies entails an increasing demand for fluorescent dyes meeting strictly defined criteria. The fluorophore motif incorporated in the structure of these molecules allows their potential use as biomarkers, fluorescent probes, laser dyes, or in the construction of organic light-emitting diodes. In the context of biomedical applications, the most valuable fluorescent dyes display absorption/emission bands shifted into the near-infrared range. Those so-called NIR dyes are used in numerous medical imaging techniques, such as FACS (Fluorescence-Activated Cell Sorting) [1,2,3], FLIM (Fluorescence-Lifetime Imaging Microscopy) [4,5,6], or FIGS (Fluorescence Image-Guided Surgery) [7,8,9]. Fluorescent dyes can be also used in imaging techniques benefiting from the two-photon absorption phenomenon [6,10,11,12,13]. In particular, special attention is paid to two-photon scanning laser microscopy, which became a breakthrough in bioimaging techniques [14,15]. Currently, the effort of many research groups is directed towards designing fluorescent dyes for specific applications, e.g., for monitoring the biological functions of organs [16,17,18], the detection of ions in living organisms [19,20,21], or for the analysis of the causes of neurodegenerative diseases [22,23,24,25].

**Figure 1:**
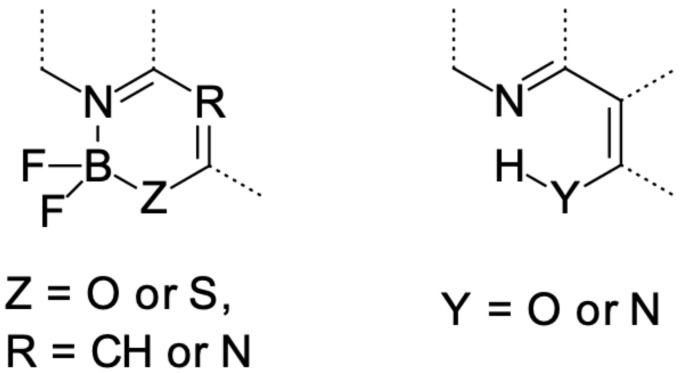
Structural motifs of difluoroboranes (**left**) and hydroxyimidazopiridine (**right**) derivatives.

There are numerous classes of fluorescent dyes, but difluoroboranes (see Figure 1, left panel) are one of the most widely employed because of their exceptional photophysical properties, such as a remarkably high fluorescence quantum yield and photostability. Their additional advantage is the ease of functionalization. Owing to the above properties, difluoroboranes have been used as optical switches [26,27,28,29,30,31], fluorescent probes [32,33,34,35,36,37,38], photosensitizers used in photochemotherapy [39,40,41,42,43,44,45], biomarkers [46,47,48,49,50], and TADF (Thermally-Activated Delayed Fluorescence) materials used for emitters for organic light-emitting diodes [51,52,53,54], among others.

Hydroxyphenylimidazopiridine derivatives (hereafter referred as HPIPs, (see Figure 1, right panel)) are yet another class of unique dyes because they can show ESIPT (Excited-State Intramolecular Proton Transfer) in both polar and nonpolar solvents, and what is even more distinctive, is that ESIPT-related emission occurs from the stable zwitterion form. Another interesting feature that some of these dyes present is so-called dual emission, in other words, the ability to simultaneously emit from two different conformers/tautomers (in two different spectral ranges). ESIPT dyes are particularly promising candidates for medicine [40,55,56], fluorescent probes [57,58,59], molecular logic gates [60,61,62], chemical sensors [63,64,65], photostabilizers [66,67,68], laser dyes [69,70,71,72] and white light-emitting material [73,74,75,76] applications, since they are characterized by unusually large Stokes shifts (exceeding even 5000 ${\mathrm{cm}}^{-1}$). This is indeed an extremely desirable feature for fluorescent dyes, as it leads to negligible overlapping of absorption and emission spectra, which in turn, prevents the emitted photons from being reabsorbed, a phenomenon that often plagues the applications of emissive dyes in biological media as well as in materials.

It should be highlighted that electronic structure theories can provide valuable insight into the absorption and emission of radiation, enabling a deep understanding of excited-state properties [77,78,79,80,81,82], which are of pivotal importance for applications in many technological sectors. Indeed, the calculations of the excited-state properties are performed not only to gain insight into absorption, fluorescence, and phosphorescence processes, but also to model the fundamental aspects of energy conversion phenomena, such as those exploited in solar cells [83,84,85,86,87,88,89]. In particular, electronic structure calculations may play a key role in tailoring the specific properties of difluoroborane and HPIP dyes needed for the above applications, provided accurate approaches are used. Undoubtedly, time-dependent density functional theory (TD-DFT) is the most widely used theoretical approach enabling computation of the optical signatures and excited-state properties of dyes [81,90,91,92,93,94,95,96]. Owing to N${}^{4}$ scaling, at first glance, it seems a cost-effective choice for medium- and large-sized dyes such as the ones mentioned above. However, the predictive power of popular density functional approximations in determining excited-state properties for these two interesting classes of dyes is largely unknown, therefore this study aims at filling this gap. Originally, the TD-DFT method was proposed by Runge and Gross [97]. However, the true milestone for practical applications was the famous Casida’s equation, developed 10 years later [98]. Introducing this effective linear-response formalism into TD-DFT granted a significant advancement of efficiency, making it possible to solve TD Schrödinger equations for many compounds. Unceasing efforts are made towards additional performance improvements and expanding the applicability of this method. Nevertheless, the predictive power of TD-DFT in terms of evaluating the properties of molecules in their excited states largely depends on the selected exchange-correlation functional and the nature of the excited state itself ($\pi \to \pi^{*}$, $n\to \pi^{*}$, charge transfer, double excitation, valence, Rydberg, singlet, triplet, etc.). To date, none of the proposed functionals can satisfactorily anticipate a wide number of properties. In this context, the true breakthrough was the introduction of so-called range-separated hybrids (RSHs), which, unlike the previously known LDA, GGA, and mGGA DFAs, are functionals including a certain fraction of the exact exchange energy related to the distance between electrons. RSHs offer significantly improved accuracy for such troublesome cases as the description of charge-transfer (CT) states [99,100,101,102,103,104]. The reason behind the significant enhancement of estimations associated with CT states lies behind employing Ewald’s split of the Coulombic electron-electron interaction operator, allowing for the introduction of the dependency of the EEX contribution on the distance between electrons. Consequently, such an approach enables introducing the correction of long-range interactions (directly related to a proper description of CT states). This scheme was proposed by Tawada, Hirao and co-workers [101] and practically utilized by Yanai, Tew and Handy who developed the most known RSH functional—CAM-B3LYP [105]. Other popular RSH functionals are frequently denoted as “LC-” (long-range corrected) DFAs, i.e., LC-BLYP [106] and LC-ωPBE [107], while another popular subgroup consists of Head-Gordon’s ωB97 functionals [108,109]. The second major step was proposing so-called double hybrids [110,111,112,113], which allows to mitigate the other known TD-DFT setback, namely, the characterization of states with a double-excited nature. In particular, these DFAs include the dependency of the exchange correlation energy of DFA on virtual Kohn-Sham orbitals, where the exchange and correlation energy terms encompass a certain fraction of the Hartree-Fock exchange, and the correlation is additionally mixed with the perturbative second-order correlation part [111]. Unfortunately, CIS(D)-like correction implies a significant increase in the computational requirements for medium and large molecules.

As can be seen, getting insight into excited-state properties exploiting TD-DFT might be quite a challenge. Therefore, to estimate the accuracy of TD-DFT predictions, one should depend on one of the two existing validation schemes described below. These validation methods are clearly important because most of the DFAs have been designed and parameterized to reproduce the properties of the ground state, not the excited state. Thus, not all DFAs are suitable for reproducing the optical properties of molecules, which in turn, significantly limits their field of application.

The first validation approach benefits from the available experimental data. Indeed, the correct reproduction of experimental data is clearly desirable for most practical applications (i.e., comparison of the experimentally determined Absorption-Fluorescence Crossing Point, AFCP with computed E${}_{0-0}$). However, in the case of the assessment of computational methods, this approach is not always optimal, since error compensation can occur due to a number of effects (i.e., interaction of the molecule with the environment, vibrational effects, etc.), thus leading to accidental agreement with the experimental data. In addition, some experimental data, like excited-state dipole moments can only be deduced indirectly (i.e., by studying solvatochromic effects and using simplified models to extrapolate results), while spectra (absorption and/or emission) in the gas phase are rarely available, meaning their use as an “absolute” benchmark is rather questionable.

The second validation scheme involves comparing TD-DFT results with predictions of more sophisticated ab initio methods, typically based on the wave function, e.g., EOM-CCSD [114,115], CAS-SCF/CAS-PT2 [116,117], or CC3 [118,119,120], which allow to describe the electronic structure of chemical compounds with high precision. Unfortunately, the application of these methods is quite restricted, on one hand by the relatively small molecular systems size (<20–30 atoms), since the price of their high accuracy is a tremendous calculation cost, and on the other hand by the quite limited accessibility of the key properties [121]. Hence, whenever the subjects under study are larger systems and/or electron-density related properties, the list of feasible methods basically shortens to ADC(2) [122,123], or CC2 [124,125]. Both aforementioned methods are characterized with typical errors within the range of 0.1–0.2 eV, which is very reasonable taking into account their $N^{5}$ scaling [121]. Importantly, a comparison with ab initio methods allows to correlate the data obtained by applying an identical computation model (e.g, exact geometry, basis set, environmental model, etc.) to the physical phenomenon.

Certainly, the excitation energy is the most common property used to benchmark TD-DFT. Within the schemes enabling the calculation of excitation energy, two different approaches should be highlighted. The first one, which is still the most common when large molecules are studied, encompasses the calculation of so-called vertical energy, E${}_{\mathrm{vert}}$, for which the computational cost is very low, since it does not require the examination of the excited state. In terms of benchmarking TD-DFT predictive power, E${}_{\mathrm{vert}}$ can be freely compared with values obtained with other, i.e., more sophisticated quantum-chemical methods; however, it does not correspond to a real absorption process, by means of the absorption maximum, $\lambda_{\mathrm{abs},\max}$. Nevertheless, E${}_{\mathrm{vert}}$ may be a very useful tool, when designing/examining a homologous series of dyes is considered. The second procedure—the adiabatic approach—allows to compute vibrationally resolved optical spectra (at least under harmonic approximation), where the computed adiabatic energy, E${}_{0-0}$, corresponds to the absorption-fluorescence crossing point, AFCP. This scheme allows for a somewhat meaningful correlation between theoretical predictions and experimentally determined data, although, as was underlined above, the straightforward evaluation of the predictive power of DFAs based on experimental data is rather disputable. Another issue is the inclusion of environmental effects into the computation, which requires the choice of a proper solvatation model, i.e., the selection of the equilibrium model is justified when the ground-state or relaxed excited-state dipole moments are evaluated; however, this model does not reflect the physics of the ultrafast absorption process. Thus, a proper examination of the Franck-Condon region of the excited state requires a nonequilibrium solvatation model. To date, many excellent studies concerning TD-DFT applicability in calculating excited-state properties can already be found in the literature. The most prominent ones enclosing benchmarks and reviews regarding the excitation energy, have been provided in the past years by Jacquemin and co-workers [94,126,127,128,129] as well as Caricato [130], Thiel [131], Grimme [132,133], Tozer [134] and Truhlar [135,136,137].

The dipole moment is a quantitative measure of the distribution of electron density, thus its change as a result of radiation absorption is directly related to the nature of the electron transitions observed (e.g., a large change in the dipole moment during electron excitation may indicate that intramolecular charge transfer occurs). In addition, the magnitude of the dipole moment also defines the strength of the molecule’s interaction with other molecules, as well as with an external electric field. Hence, the electrical properties of the molecules, such as their dipole moment or polarizability, ensure one of the most direct relationships between the electronic structure of the molecules and the spectroscopically observed quantities, making them the right properties to assess the accuracy of computational methods. It should be emphasized that, unlike electron density, which is a function of coordinates in the selected coordinate system, the dipole moment is an easy to calculate property and convenient to evaluate the predictive power of DFAs in assessing asymmetry in the electron density distribution. Despite the ease of this computational routine, the number of extensive dipole moment ($\mu_{\mathrm{GS}}$ or/and $\mu_{\mathrm{ES}}$ or/and the excess dipole moment) benchmarks is rather scarce [100,138,139,140,141,142,143,144]. It should be stressed that although in the literature one may find a fair number of works focusing on the estimation of excitation energy as well as some studies on dipole moments, the subjects of the vast majority are ‘model’ small organic compounds. What is even more striking, the number of investigated molecules in most works rarely exceeds 30, usually highly specific compounds, whereas the number of included DFAs scarcely ever exceeds three.

Another subject to tackle is the constant development of new functionals, which entails the necessity of assessing their predictive power and comparing them with other already known DFAs.

As can be seen from the above literature survey, there are still some blank pages to be filled. In particular, as already highlighted in the preceding paragraph, the predictive power of popular density functional approximations in predicting excited-state properties for HPIPs and difluoroboranes is largely unknown, which prohibits using TD-DFT with confidence for designing new derivatives with tailored properties. Hence, to overcome these limitations, this study focuses on a large set of molecules encompassing 85 ‘real-life’ dyes originating from two important classes of fluorescent dyes, difluoroboranes and HPIPs. The common denominator of the above dyes is a D-π-A structural core. The first set benefits from incorporating a BF${}_{2}$ group which stabilizes the six-membered ring, while the latter set introduces intramolecular hydrogen bonding enabling ESIPT. Described fluorescent dyes are investigated using 18 DFAs that can be divided into functionals without EEX (e.g., BLYP, M06-L), with a small contribution of EEX (e.g., BLYP, PBE0), with a major fraction of EEX (e.g., MN15, M06-HF) and RSHs (e.g., CAM-B3LYP, ωB97X-D). In more detail, the unique aim of this work is to provide reliable data regarding the evaluation of the predictive power of DFAs in terms of the two most important excited-state properties (excitation energy and dipole moments), facilitating the modeling of fluorescent dyes. Since identical geometries and theory levels were used for vertical energy and dipole moment calculations, the presented data allow to directly define the relation between the DFA accuracy in estimating energetic and electron density-related properties. This is particularly important, considering that the only other TD-DFT study dedicated to benchmarking vertical energy and dipole moments on the very same theory level was done by Silva-Junior, Thiel and co-workers [131,145]. It should be mentioned that within the chosen set, several dyes exhibit charge transfer during excitation, which is a well-known issue of the TD-DFT approach. Within the chosen DFA set, there are MN15 and APF-D, which were the objects of very few benchmarks/reviews previously [135,146,147], thus their accuracy in predicting the spectroscopic properties of fluorescent dyes is yet to be determined.

## 2. Materials and Methods

All DFT and TD-DFT calculations were performed using the Gaussian 16 (B.01) program package [148], whereas CC2 calculations were executed using the Turbomole code [149]. Firstly, all 85 structures were optimized in the gas phase using B3LYP [150] and the cc-pVDZ basis set [151]. The optimization threshold was improved to 10${}^{-5}$ a.u. on average residual forces, whereas a self-consistent field convergence criterion was tightened to $10^{-8}$ a.u. In the above computations, the so-called ultrafine-pruned (99,590) integration grid was applied. For all the examined structures, Hessian calculations confirmed that the designated stationary points are the actual minima on the potential energy hypersurface. In the next step, the optimized structures were used to calculate the electronic structure to determine the spectra of one-photon absorption and dipole moments in the ground state and the Franck-Condon region. Excitation energies as well as dipole moments were determined using 18 different correlation-exchange functionals (SVWN [152,153], BLYP [150,154], TPPSh [155], B3LYP [156], X3LYP [157], CAM-B3LYP [105], LC-BLYP [106], APF-D [158], PBE0 [90,159], M06 [137], M06-L [160], M06-HF [161], M06-2X [137], SOGGA11-X [162], BMK [163], MN15 [135], ωB97X [109], ωB97X-D, [109]) and the aug-cc-pVDZ basis set. In the last step the above data was compared with the reference results from the RI-CC2 method (obtained with the aug-cc-pVDZ [151] basis set using the recommended auxiliary function database [164]).

Among the studied functionals, we highlight three of them as particularly important for this benchmark study:

MN15 is the first global hybrid meta-nonseparable gradient approximation (NGA), proposed by the Truhlar group. As the name suggests, in contrast to standard XCF, this class of functionals does not rely on the partition of energy into exchange and correlation terms. It is a hybridized version of the local MN15-L functional with 58 parameters. Owing to the training on an extended dataset including a wide range of DFT-challenging properties, the above DFA offers not only a good performance for noncovalent interactions and excitation energies, but also improved energies of multireference systems and barrier heights [135].

SOGGA11-X is a 19-parameter variant of SOGGA11 including 40.15% of Hartree-Fock exchange, proposed by Peverati and Truhlar. It is the first global hybrid GGA offering correct estimation to the second order, for both exchange and correlation terms; however, since it lacks dependence on the kinetic energy density, it is outperformed by hybrid meta-GGAs, like M06 and M11 [162].

ωB97X-D is a range-separated hybrid (X = 22.2–100) GGA including empirical atom–atom dispersion corrections developed by Head-Gordon and Chai [108]. This 18-parameter functional usually provides satisfactory accuracy for thermochemistry, kinetics, and non-covalent interactions. Owing to reoptimization, ωB97X-D offers a significantly improved performance for noncovalent interactions over its predecessor, ωB97X.

## 3. Results and Discussion

Below, we present the results of the calculations performed for the lowest excitations of the isolated molecules (see Figures S1–S3), which were carried out to determine the DFA with the highest predictive power, for both the vertical energy and dipole moments ($\mu_{\mathrm{GS}}$ and $\mu_{\mathrm{ES}}$). The electron density difference plots (together with the charge-transfer diagnostic [91]) predicted with the chosen subset of DFAs for exemplary charge-transfer and local states, are available in ESI Tables S3–S6. All structures of difluoroboranes included within this study were synthesized by the Ośmiałowski group [165,166,167,168,169], while the HPIP set consists of structures reported by Acuña [170], Gryko [171,172,173,174], Cossio [175], Araki [176], Shimada [177] and Bajipali [178]. Since, in compounds stabilized by an intramolecular hydrogen bond, proton transfer in the ground state is still possible, it is worth underlining that the all structures shown in SI are stable, ground-state forms. Lastly, it should be emphasized that BF${}_{2}$ carrying molecules are rigid ones with easily visible vibrational features in their absorption and emission spectra [165,168], while for the HPIP family of dyes these are weakly visible [172]. The current benchmark thus encompasses compounds of different rigidity.

To enable a straightforward analysis, all results are presented in the form of various errors (i.e., mean absolute error (MAE), maximum absolute error (MAXAE), root mean square error (RMSE)) and the correlation coefficient $R^{2}$, all determined with respect to the reference method (CC2). Notably, for the sake of a reduction in the computational costs, the C${}_{8}$H${}_{17}$ substituents attached to some of the extended-HPIPs [174] (i.e., 1-HPIP and 28-HPIP, see ESI Figures S2 and S3), have been replaced with methyl groups. It should be stressed, that the choice of CC2 as a reference method was dictated by the considerable size of the examined fluorescent dyes, as well as the implementation of the analytical calculation of the electron density, enabling the computation of excited-state dipole moments. The latter one is particularly important, since the goal of this study is to provide coherent estimations of DFA excitation energies together with ground-state and excited-state dipole moments. Nevertheless, the authors are fully aware that the CC2 approach is not ‘error-free’, in fact Thiel pointed out that CC2 vertical energies are quite far from TBEs (Theoretical Best Estimates) [131], nevertheless CC2 results presented a nearly linear correlation with TBEs. Thus, despite known limitations, CC2 can be regarded as a valuable tool for assessing DFA accuracy in real-life molecules, for which sophisticated ab initio methods are unavailable. Moreover, according to the recent work of Loos et. al., the typical errors of the CC2 method vary in the range of 0.1–0.2 eV [121], which nicely correspond to the MAEs (0.12 eV) reported by Thiel and Sauer [179]. In turn, Kozma et. reported much higher errors for two-component molecular complexes displaying CT states (MSE = −0.36 eV) [180]. As far as dipole moments are concerned, Loos and Jacquemin performed the evaluation of the $\mu_{\mathrm{GS}}$ of small molecules, reporting CC2 MAEs of between 0.09 and 0.12 D (depending on the chosen LR scheme), while ES dipole moments were burdened with MAEs in the range of 0.26–0.67 D [144].

### 3.1. Vertical Energies

**Figure 2:**
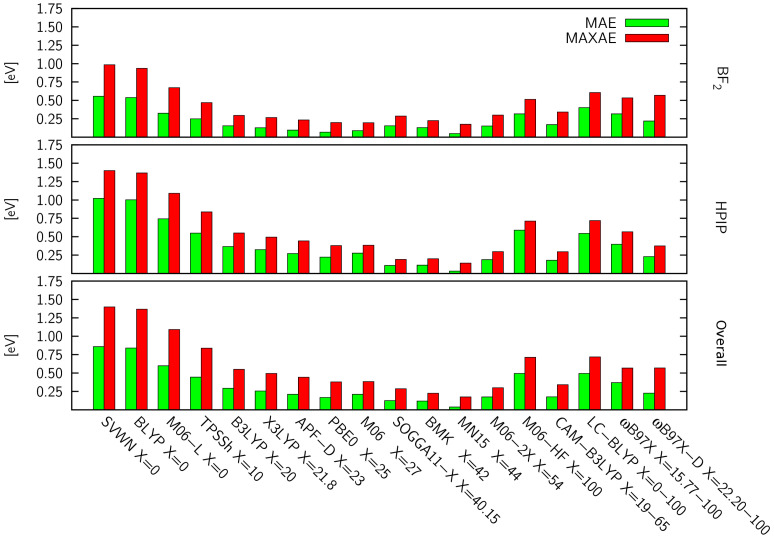
DFA absolute errors established for the excitation energy, determined with respect to CC2.

Different types of errors determined for the respective DFAs are displayed in Table 1 and Figure 2. At first glance, one can see a clear relationship between the fraction of the energy corresponding to the exact exchange and the predictive power of a DFA (see “X”, Table 1). Secondly, the error trends of individual functionals are quite similar for both groups of dyes, however, qualitatively larger errors are observed for HPIPs. As expected, among the examined set of DFAs, the largest errors are always observed for ‘pure’ functionals such as BLYP and SVWN, i.e., the MAE above 0.5 eV for BF${}_{2}$ and over 1.0 eV for HPIP compounds. It should be highlighted that the related correlation coefficients are also completely unsatisfactory ($R^{2}$ ca. 0.7). Generally, DFAs distinguished by an EEX equal to 0% or 100% i.e., all LDA and GGA as well as some RSHs, systematically underestimate the excitation energy (BLYP, M06-L and SVWN) or significantly overestimate it (M06-HF, LC-BLYP). Clearly, the errors yielded by meta-GGA or RSH functionals are less severe then the aforementioned LDA and GGA DFAs. However, even for LC-BLYP, M06-L and M06-HF, MAEs are still rather disappointing (0.3–0.4 eV and 0.5–0.75 eV for BF${}_{2}$ and HPIPs, respectively).

Among the RSH, the most accurate predictions were obtained with CAM-B3LYP, which delivered MAEs not exceeding 0.18 eV, while the MAXAEs reached, at most, 0.35 eV for both BF${}_{2}$ and HPIP derivatives. Data presented in Figures S4 and S5 show that CAM-B3LYP overestimates (usually < 0.2 eV) reference excitation energies for both BF${}_{2}$ and HPIP derivatives. Much higher errors concerning the excitation energy were generated by LC-BLYP, which systematically overestimates the excitation energy, i.e., MAEs reaching 0.4 and 0.5 eV, respectively for BF${}_{2}$ and HPIPs. This research also included some RSHs designed by Head-Gordon et al., namely, ωB97X [109] and ωB97X-D [108]. Both of the aforementioned DFAs overestimate the excitation energy, however the errors provided by ωB97X-D (MAE ≈ 0.20 eV for both dye sets) are clearly lower than for the ωB97X counterpart. Interestingly, CAM-B3LYP and ωB97X-D are among the few DFAs that managed to predict the excitation energies of both dye groups with similar accuracy. APF-D is a functional included in this series that is rarely used to study electronic excited states. Surprisingly, this DFA provides very accurate predictions of the excitation energies of BF${}_{2}$ dyes, with the MAE of only 0.09 eV and a high $R^{2}$, which is the second best result for this series. Nevertheless, the predictions of HPIP excitation energies are burdened with an almost three-times greater MAE (0.27 eV) and a significantly lower correlation coefficient ($R^{2}$ = 0.90), while the MAXAE exceeds 0.40 eV.

The most extensive group tested here are hybrid DFAs benefiting from the constant fracture of EEX. For the purposes of this work, they were divided into two subgroups, DFAs containing a minor fraction of EEX (below 30%, i.e., M06-L, TPSSh, B3LYP, X3LYP, PBE0, M06) and those encompassing a major contribution of EEX (above 40%, i.e., SOGGA11-X, BMK, MN15, M06-2X, M06-HF). In terms of DFAs incorporating the minor EEX contribution, the most underestimated excitation energies were predicted by TPSSh including a small EEX contribution (10%). Results displayed in Figures S4 and S5 show that in general, hybrid DFAs incorporating a 20%–30% EEX fraction (i.e., B3LYP, X3LYP, PBE0, M06), tend to underestimate the excitation energy (ca. 0.1–0.2 eV for the BF${}_{2}$ set and 0.2–0.4 eV for HPIPs). Consequently, identical trends are observed for MAEs, i.e., significantly higher values are provided for HPIPs (in the range of 0.22–0.37 eV), than for BF${}_{2}$ dyes (0.07–0.15 eV). It should be highlighted that the above DFAs deliver very consistent results with a strong linear dependence, i.e., $R^{2}$ oscillating around 0.95 and RMSE in the range of 0.02–0.04 eV for BF${}_{2}$ dyes. Conversely, the mentioned parameters corresponding to HPIPs are considerably worse ($R^{2}$ fluctuating around 0.90 and RMSE exceeding 0.10 eV). Independently of the dye data set, the best results among this group of DFAs were achieved with PBE0, although MAE and MAXAE related to HPIPs are respectively, two-times and three-times higher than their BF${}_{2}$ counterparts.

The data presented in Figure 2 and ESI Figures S4 and S5 clearly indicate, that regardless of the tested dye series, the most accurate excitation-energy predictions are offered by DFAs containing a 40–55% EEX fraction, i.e., SOGGA11-X, BMK, MN15 and M06-2X. It is worth noting that aside the aforementioned CAM-B3LYP and ωB97X-D, these are the only DFAs generating quantitatively similar errors for the BF${}_{2}$ and HPIP sets. Most DFAs consisting of 40–55% EEX, overestimate the excitation energy by ca. 0.1–0.2 eV (except MN15). Consequently, MAEs produced by these DFAs do not exceed 0.2 eV (MAXAEs < 0.3 eV), regardless the dye set. Notably, the results of this subgroup of DFAs present a high correlation with the reference for both dye groups ($R^{2}$≥ 0.94); however, the RMSEs for HPIPs are ca. two-times higher than their BF${}_{2}$ analogs. Within the 18 functionals examined in this work, the most accurate estimations of excitation energies were provided by the relatively new “Minnesota” family DFA, namely, MN15. This DFA generates the MAE of 0.05 eV for BF${}_{2}$ and only 0.03 eV for HPIP derivatives, while the MAXAEs stay below 0.2 eV. Notably, MN15 provides the most consistent results among the examined series, with a $R^{2}$ of 0.95 (0.97) and RMSE equal to 0.01 eV (0.03 eV) for BF${}_{2}$ (HPIP) dyes. In turn, the most overestimated results within DFAs with a major EEX contribution, were provided by M06-HF (EEX = 100%), i.e., MAE = 0.31 eV for BF${}_{2}$ and MAE = 0.59 eV for HPIPs. Besides that, M06-HF is characterized by a lower $R^{2}$ (<0.95) and RMSE (≥0.09 eV) than other functionals in this subgroup.

**Figure 3:**
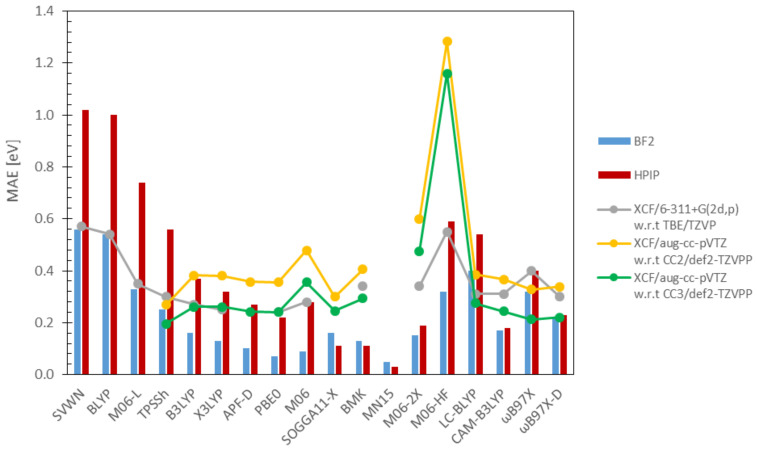
Comparison of DFAs predictive power; data presented in columns concern vertical energies calculated in this work, while the information displayed as “lines” relates to literature data—grey is the Thiel set; yellow, 104 singlet ES [126]; and green, 41 ES of small- and medium-sized organic molecules [129].

Lastly, the presented results are in line with previous studies available in the literature. Figure 3 depicts the comparison between MAEs found for fluorescent dyes examined herein and the results reported by Jacquemin and co-workers [126,129]. The aforementioned studies were chosen due to their provision of statistically meaningful data concerning accuracy (i.e., the vast number of examined excitations and a large set of DFAs) as well as comparable (ab initio) reference methods, which enables a straightforward comparison. As can be seen, the error profiles of the compared studies bear many resemblances, although substantial quantitative discrepancies are observed for M06-HF, i.e., the MAE determined with respect to the TBE/TZVP is comparable to the one generated for the HPIP set, while the errors established with respect to the CC methods are almost two-times higher.

A separate matter worth addressing is the accuracy of CC2 (chosen as the reference method herein), as shown by Jacquemin, CC2 delivers qualitatively consistent results with CC3, since both error profiles (see yellow and green lines in Figure 3) have an almost identical shape. This proves that CC2 indeed can be used as the reference method for systems too complex for more accurate methods, like CC3. According to the authors best knowledge, the only extensive study concerning MN15 was published in 2016 by the Truhlar team [135]. They reported that MN15 generates the MAE equal to 0.29 eV for the set of valence states included in the EE69 database, which is a considerably higher error than the ones presented within this work.

### 3.2. Dipole Moments

**Figure 4:**
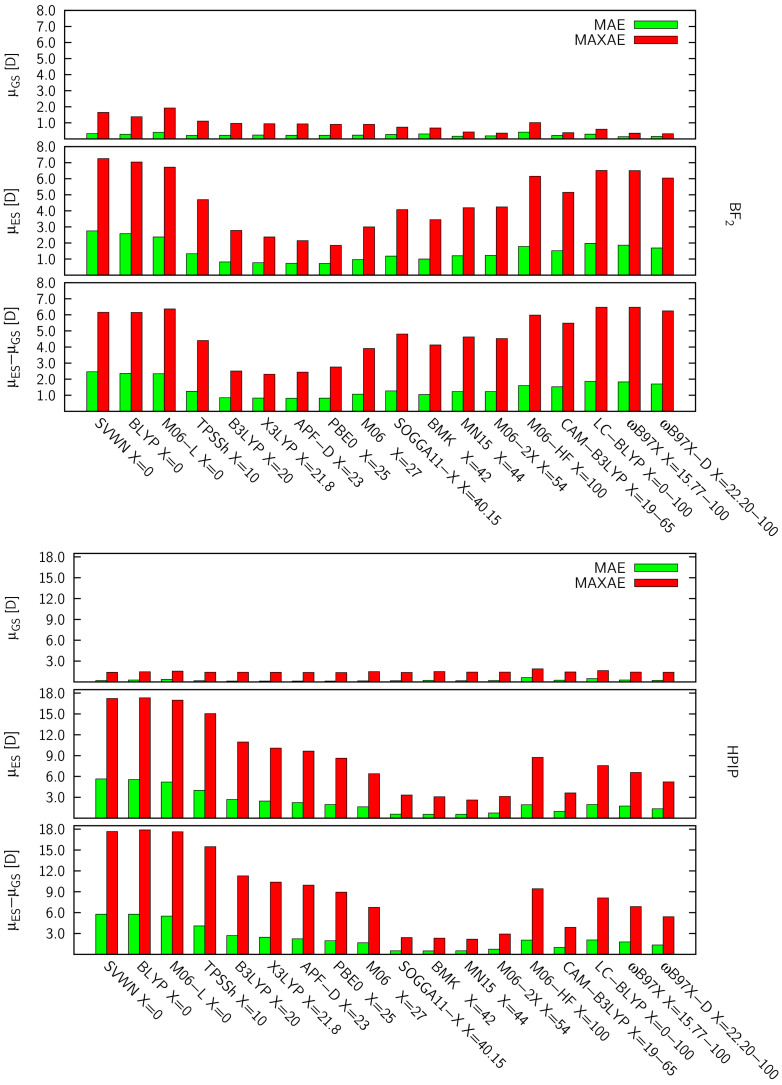
DFA errors established for the dipole moments (determined with respect to CC2).

All data concerning dipole moments are collected in Table 2 and Figure 4. For the sake of avoiding any confusion, the results concerning $\mu_{\mathrm{GS}}$ and $\mu_{\mathrm{ES}}$ (together with the excess dipole moment, Δμ=$\mu_{\mathrm{ES}}-\mu_{\mathrm{GS}}$) will be discussed separately.

#### 3.2.1. Ground-State Dipole Moments

Generally, DFA predictions of $\mu_{\mathrm{GS}}$ are burdened with rather small MAEs i.e., for both BF${}_{2}$ and HPIP derivatives, the highest errors were generated by M06-HF (MAEs exceeding 0.4 D). However, in terms of the MAXAE, M06-L introduces the highest errors (BF${}_{2}$ ca. 2.0 D, HPIP ca. 1.6 D), which in turn manifests in a significantly lower correlation coefficient for BF${}_{2}$ dyes, i.e., $R^{2}$ = 0.82. Those observations are quite expected since the above ‘Minnesota’ DFAs, M06-L and M06-HF, are characterized with an EEX = 0% and 100%, respectively; however, one would anticipate an even weaker accuracy of BLYP and SVWN functionals. Interestingly, BLYP and SVWN predictions present slightly better accuracy and consistency for the HPIP set (MAE ≈ 0.25 D, RMSE ≈ 0.05 D) than BF${}_{2}$ dyes (MAE ≈ 0.30 D, RMSE > 0.1 D).

In turn, all DFAs including 20%–30% EEX (B3LYP, X3LYP, APF-D, PBE0, M06) are characterized with a rather similar performance among each set of dyes, i.e., MAE values around 0.23 D and 0.10 D for BF${}_{2}$ and HPIP, respectively. Consequently, the correlation coefficients and RMSEs of these DFAs are the subjects of equally subtle variations. As mentioned above, MAEs related to HPIPs are almost two times lower than the BF${}_{2}$ counterparts, however a reverse trend is observed for MAXAEs. It should be emphasized that the very small differentiation among DFA $\mu_{\mathrm{GS}}$ results, prevents a reliable identification of trends, and thus selection of the best DFA among this subgroup.

In contrast to DFAs containing a minor EEX fraction, results delivered by DFAs incorporating 40%–55% EEX (BMK, SOGGA11-X, MN15, M06-2X) differ substantially. For BF${}_{2}$ molecules, BMK and SOGGA11-X predictions bear similar accuracies (MAE ≈ 0.3 D, MAXAE ≈ 0.7 D), whereas considerably lower errors are obtained with MN15 and M06-2X (MAE ≈ 0.2 D, MAXAE ≈ 0.4 D). On the other hand, MAE (MAXAE) errors generated for HPIPs span across 0.14–0.20 D (1.29–1.42 D) range. Notably, for both dye groups, the smallest MAEs were generated by MN15, which together with M06-2X and CAM-B3LYP, are the only DFAs in the examined series providing similar and yet satisfying accuracy for both BF${}_{2}$ and HPIP dyes.

Among the RSH functionals, the highest mean and maximum errors are related to LC-BLYP (EEX = 0–100%) predictions, independently of the examined dye group. In turn, better accuracy is achieved by CAM-B3LYP, which provides almost identical MAEs for HPIP and BF${}_{2}$ (ca. 0.20 D). For BF${}_{2}$ dyes a superior performance is achieved by ωB97X and ωB97X-D DFAs, with a slightly lower mean error generated by ωB97X (MAE = 0.15 D, MAXAE = 0.36 D). While a reverse trend is observed for HPIPs, i.e., ωB97X-D provides the most accurate prediction among the RSH functionals (MAE = 0.2 D, MAXAE = 1.3 D).

The above trends show clearly that one should be careful in choosing the DFA even for GS parameter calculations. Nevertheless, all DFAs are characterized by very high consistency factors, i.e., $R^{2}$ > 90 (except M06-L, BF${}_{2}$) and RMSEs rarely exceeding 0.1 D.

**Figure 5:**
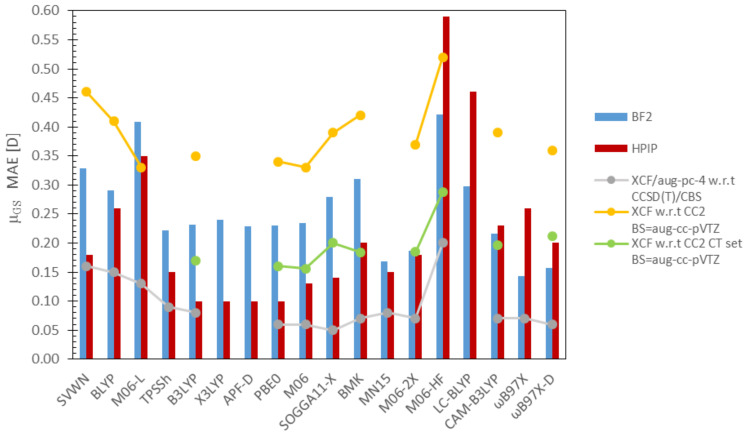
Comparison of DFAs predictive power; data presented in columns concern $\mu_{\mathrm{GS}}$ calculated in this work, while information displayed as “lines” relate to literature data—grey, the database of 200 dipole moments of small inorganic molecules developed by Gordon and Hait [143]; yellow, a set of 15 small organic molecules; and green, 15 large organic molecules exhibiting CT [142].

In the literature there are available extensive data treating $\mu_{\mathrm{GS}}$; however, as can be seen from Figure 5, their DFA sets only partially coincide with this study. The most extensive work concerning 200 $\mu_{\mathrm{GS}}$ was presented by Hait and Gordon; however the subjects of the mentioned study are small inorganic compounds [143], thus, obviously their $\mu_{\mathrm{GS}}$ values are significantly smaller (see grey line, Figure 5) than the ground-state dipole moments of fluorescent dyes reported here. Nevertheless, one can observe similarities between the error line presented by the Gordon series and the error bars related to the HPIP set; however, the trends for pure DFAs are completely opposite. In the context of this work, more relevant data were reported by Jacquemin, who examined $\mu_{\mathrm{GS}}$, $\mu_{\mathrm{ES}}$ and excess dipole moments of 30 challenging medium and large organic molecules, dividing them into two series based on the nature of the excitation [142]. As can be seen from Figure 5, DFA predictions computed for BF${}_{2}$ and HPIPs are closely related to the series bearing molecules with a strong CT nature (green line).

#### 3.2.2. Excited-State and Excess Dipole Moments

Data collected in Table 2 and Figure 4 show clearly that the predictions of $\mu_{\mathrm{ES}}$ (in the Franck-Condon region) are burdened with much larger errors than the corresponding $\mu_{\mathrm{GS}}$, i.e., for most of the investigated DFAs the difference between $\mu_{\mathrm{ES}}$ and $\mu_{\mathrm{GS}}$ errors exceeds one order of magnitude. Of course, this trend was expected, since a large number of the DFAs are parameterized to properly evaluate ground-state properties, not the excited state. Clearly, the magnitude of the $\mu_{\mathrm{ES}}$ errors directly determines the scope of excess dipole moments corresponding to the absorption transition. Consequently, the error profiles of $\mu_{\mathrm{ES}}$ and the related Δμ displayed in Figure 4, are almost identical. At this point, it should be highlighted that $R^{2}$ and RMSE corresponding to excess dipole moments are directly related to the accuracy of the $\mu_{\mathrm{GS}}$ and $\mu_{\mathrm{GS}}$ components; thus, their straightforward analysis is rather questionable.

As expected, the predictions of pure DFAs (without EEX contribution), i.e., BLYP, SVWN, and M06-L, are burdened with the most severe errors (slightly lighter for M06-L). The above DFAs generate MAEs two-times higher for HPIP (in 5.2–5.6 D range) than BF${}_{2}$ dyes (2.2–2.6 D), and even greater discrepancies are observed for MAXAEs, i.e., BLYP, SVWN and M06-L can overestimate $\mu_{\mathrm{ES}}$ for HPIPs by nearly 20 D (!). Notably, the low correlation factors (0.36–0.45) and high RMSE values (above 4 D) obtained for the HPIP set demonstrate, that DFAs without EEX are clearly among the worst possible choices for the calculation of density-related properties of challenging molecules exhibiting CT. In turn, the $\mu_{\mathrm{ES}}$ results obtained for BF${}_{2}$ dyes are characterized with $R^{2}$ factors comparable to their $\mu_{\mathrm{GS}}$ counterparts; however, RMSE values related to ES calculations are clearly much higher.

Among all examined DFAs, functionals including a 20–30% EEX fraction (B3LYP, X3LYP, APF-D, PBE0, M06) predict the $\mu_{\mathrm{ES}}$ of BF${}_{2}$ molecules with greatest accuracy, i.e., MAE values vary in the the range 0.75–0.97 D, while MAXAEs do not exceed 3.0 D. Additionally, those DFAs provide very consistent results (RMSE < 0.4 D for $\mu_{\mathrm{ES}}$ and excess dipole moments) highly correlated with the reference ($R^{2}$≥ 0.92). Amid DFAs containing a minor EEX fraction, the best results were delivered by APF-D and PBE0 (MAE ≈ 0.7 D), however, PBE0 generated a slightly lower MAXAE (<2.0 D). In turn, predictions of these DFAs for HPIPs are burdened with a MAE ca. three-times higher (except M06), together with an increased RMSE (1.0–2.0 D except M06) and considerably lower $R^{2}$ (0.65–0.86) values. As can be seen, for HPIP dyes, M06 offers considerably better accuracy of $\mu_{\mathrm{ES}}$ values (MAE = 1.60 D, MAXAE = 6.4 D), than the other examined DFAs with minor EEX contributions.

On the other hand, the smallest deviations for HPIPs were obtained with DFAs containing 40–55% EEX (SOGGA11-X, BMK, MN15, M06-2X). Indeed, SOGGA11-X, BMK, and MN15 include very similar EEX fractions (40–44%), which explains their highly coinciding results, i.e., MAEs oscillating around 0.53 D and MAXAEs in the range 2.2–2.4 D. Accordingly, M06-2X including a slightly higher EEX contribution (54%) generates slightly increased errors (MAE = 0.7 D, while MAXAE exceeds 3 D). Notably, the above functionals provide the best correlation factors ($R^{2}$≈ 0.9) and the lowest RMSEs (below 0.3 D) for HPIPs among all examined DFAs. Similar trends can be extrapolated to BF${}_{2}$ dyes; however, the accuracy of the mentioned DFAs is somewhat lower, i.e., MAEs in the range 1.0–1.2 D and MAXAEs encompassing a 3.5–4.2 D range.

In turn, M06-HF (EEX = 100%) predictions for the HPIP set bear a very low $R^{2}$, which implies there is no linear correlation between the M06-HF results and reference values whatsoever. The identical conclusion can be drawn for LC-BLYP estimations of the HPIPs excited-state dipole moments.

Lastly, considering RSHs (LC-BLYP, CAM-B3LYP, ωB97X, ωB97X-D), the most accurate results for the both sets of dyes are provided by CAM-B3LYP; however, HPIP predictions are burdened with considerably lower errors (MAE = 0.95 D, MAXAE = 3.62 D). Unexpectedly, ωB97X and ωB97X-D present rather unsatisfactory $R^{2}$ values (0.3 and 0.6, respectively) conjoined with high RMSEs (1.0 D and 1.8 D) for HPIPs, while in comparison their BF${}_{2}$ counterparts display a $R^{2}$ of ca. 0.95 and RMSE below 0.7 D.

**Figure 6:**
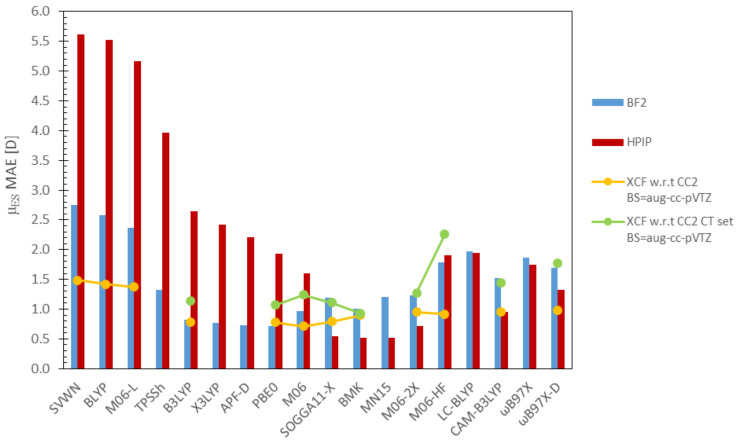
Comparison of DFAs predictive power; data presented in columns concern $\mu_{\mathrm{ES}}$ calculated in this work, while information displayed as “lines” relate to literature data—yellow, a set of 15 small organic molecules and green, 15 large organic molecules exhibiting CT [142].

Literature data used for the comparative analysis are taken from Jacquemin’s study mentioned in the preceding paragraph. As can be seen in Figure 6 the highest discrepancies between the $\mu_{\mathrm{ES}}$ predictions reported here and by Jacquemin are related to DFAs without EXX (above 1 D), which are commonly known for their poor performance. Other predictions are quantitatively similar, although there is not much to be said about the error trends since six of the DFAs examined here were not investigated by Jacquemin.

## 4. Conclusions

The presented study focuses on providing extensive data about TD-DFT predictive power, evaluated for two groups of important dyes encompassing difluoroboranes and HPIP derivatives. To ensure statistically meaningful results, the data set compromises 85 molecules manifesting diverse photophysical properties. The emphasis was put on the lowest excitations which in many instances possess a charge-transfer character (see Tables S9 and S10 presenting the values of the Λ diagnostic proposed by Peach et al. [134]). The vertical excitation energies and dipole moments ($\mu_{\mathrm{GS}}$, $\mu_{\mathrm{ES}}$ and Δμ) of the aforementioned dyes were determined by 18 DFAs, with varying contributions of EEX. In particular, this study proves that DFAs incorporating 0–30% EEX tend to underestimate the excitation energy, while increasing the EEX above 50% results in a reverse trend. Interestingly, quantitatively similar errors for both dye groups are generated only by DFAs containing 40–50% of EEX and two RSHs, CAM-B3LYP and ωB97X-D. For the rest of the examined DFAs, systematically larger errors (MAE and MAXAE) are observed for HPIP derivatives. Within the tested DFA set, the most accurate vertical energy predictions were obtained with MN15 (MAE ≤ 0.05 eV), which significantly outperforms the second best functional, PBE0 (MAE = 0.22 eV for HPIPs).

A similar analysis performed for dipole moments indicates that within the examined set of DFAs, there is no single functional that would equally accurately determine GS and ES dipole moments of BF${}_{2}$ and HPIP dyes. In the case of $\mu_{\mathrm{GS}}$ the most accurate results were predicted by one of the DFAs with a major EEX fraction, namely MN15 (BF${}_{2}$ set) and the DFA with a minor EEX contribution, PBE0 (HPIP set); however, all DFAs incorporating 20–30% EEX deliver very similar predictions. On the other hand, reverse trends are observed for $\mu_{\mathrm{ES}}$, i.e., for BF${}_{2}$, the best results were obtained with DFAs including a minor fraction of EEX (B3LYP, X3LYP, PBE0, M06 and APF-D), specifically PBE0, while in the case of HPIP derivatives, much more accurate predictions were provided by DFAs with a major EEX contribution, especially MN15.

To sum up, MN15 strongly outperforms other DFAs in terms of determining the excitation energy; however, in the context of dipole moments, there is no single DFA that would equally accurately determine GS and ES dipole moments of BF${}_{2}$ and HPIP derivatives. Nevertheless, analyzing the results for the combined set of dyes (see Overall, Table 2) one can see that MN15 generates the lowest $\mu_{\mathrm{ES}}$ errors together with one of the most accurate $\mu_{\mathrm{GS}}$, therefore this functional should be dedicated to the further study of ‘real-life’ fluorescent dyes. The other conclusion that can be formulated, is that methods delivering very accurate predictions for smaller molecules can often not be adequate for examining complex real-life dyes. Thus, one should carefully choose the density functional approximation and/or think about a validation scheme suitable for their purpose.

## Figures and Tables

**Table 1:** DFA errors (in [eV]) established for the vertical energy, determined with respect to CC2 results.

Functional	X; ω	BF${}_{2}$				HPIP				Overall			
		MAE	MAXAE	$R^{2}$	RMSE	MAE	MAXAE	$R^{2}$	RMSE	MAE	MAXAE	$R^{2}$	RMSE
SVWN	0	0.54	0.98	0.70	0.31	1.02	1.40	0.49	0.72	0.86	1.40	0.24	0.79
BLYP	0	0.53	0.94	0.73	0.28	1.00	1.37	0.50	0.70	0.84	1.37	0.25	0.77
M06-L	0	0.31	0.67	0.80	0.15	0.74	1.09	0.60	0.54	0.60	1.09	0.38	0.51
TPSSh	10	0.24	0.47	0.91	0.08	0.56	0.84	0.75	0.37	0.44	0.84	0.67	0.28
B3LYP	20	0.15	0.30	0.95	0.04	0.37	0.55	0.87	0.22	0.29	0.55	0.85	0.12
X3LYP	21.8	0.12	0.27	0.96	0.03	0.32	0.49	0.89	0.19	0.26	0.49	0.87	0.10
APF-D	23 *	0.09	0.23	0.96	0.02	0.27	0.44	0.90	0.16	0.21	0.44	0.89	0.08
PBE0	25	0.07	0.20	0.96	0.02	0.22	0.38	0.92	0.14	0.17	0.38	0.91	0.06
M06	27	0.09	0.20	0.95	0.02	0.28	0.38	0.93	0.14	0.21	0.38	0.90	0.08
SOGGA11-X	40.15	0.15	0.29	0.94	0.04	0.11	0.19	0.96	0.07	0.12	0.29	0.97	0.02
BMK	42	0.13	0.22	0.96	0.03	0.11	0.20	0.96	0.07	0.12	0.22	0.98	0.02
MN15	44	0.05	0.18	0.95	0.01	0.03	0.14	0.97	0.03	0.04	0.18	0.98	0.01
M06-2X	54	0.15	0.30	0.95	0.04	0.19	0.30	0.96	0.09	0.17	0.30	0.98	0.03
M06-HF	100	0.31	0.51	0.92	0.09	0.59	0.71	0.87	0.27	0.49	0.71	0.90	0.16
LC-BLYP	0–100; 0.33	0.40	0.61	0.91	0.12	0.54	0.72	0.90	0.24	0.49	0.72	0.93	0.13
CAM-B3LYP	19–65; 0.33	0.17	0.34	0.94	0.04	0.18	0.30	0.95	0.09	0.17	0.34	0.97	0.03
ωB97X	15.77–100; 0.30	0.31	0.53	0.90	0.10	0.40	0.57	0.92	0.18	0.37	0.57	0.95	0.08
ωB97X-D	22.20–100; 0.20	0.22	0.57	0.91	0.07	0.23	0.38	0.94	0.12	0.23	0.57	0.96	0.05

* APF-D functional adopts an empirical dispersion correction method based on a spherical atom model (SAM), instead of the ‘traditional’ Grimme’s empirical dispersion correction method. See [158] for more information.

**Table 2:** DFA errors (in [D]) established for the dipole moments, determined with respect to CC2 results.

DFA	Dipole Mom.	BF${}_{2}$				HPIP				Overall			
		MAE	MAXAE	$R^{2}$	RMSE	MAE	MAXAE	$R^{2}$	RMSE	MAE	MAXAE	$R^{2}$	RMSE
SVWN	$\mu_{\mathrm{GS}}$	0.32	1.65	0.92	0.14	0.25	1.34	0.98	0.04	0.28	1.34	0.97	0.11
	$\mu_{\mathrm{ES}}$	2.62	7.25	0.94	0.82	5.61	17.23	0.36	5.11	4.61	17.23	0.58	3.59
	$\mu_{\mathrm{GS}}-\mu_{\mathrm{ES}}$	2.34	6.16	0.93	0.79	5.83	18.30	0.47	4.88	4.65	18.30	0.56	3.83
BLYP	$\mu_{\mathrm{GS}}$	0.29	1.38	0.92	0.11	0.26	1.46	0.98	0.05	0.27	1.46	0.97	0.06
	$\mu_{\mathrm{ES}}$	2.45	7.04	0.94	0.77	5.52	17.32	0.45	4.53	4.49	17.32	0.62	3.26
	$\mu_{\mathrm{GS}}-\mu_{\mathrm{ES}}$	2.23	6.15	0.93	0.76	5.76	17.89	0.60	4.02	4.58	17.89	0.62	3.35
M06-L	$\mu_{\mathrm{GS}}$	0.41	1.92	0.83	0.26	0.35	1.56	0.98	0.06	0.37	1.92	0.94	0.12
	$\mu_{\mathrm{ES}}$	2.22	6.72	0.94	0.73	5.17	16.98	0.45	4.28	4.19	16.98	0.63	3.04
	$\mu_{\mathrm{GS}}-\mu_{\mathrm{ES}}$	2.20	6.37	0.93	0.76	5.49	17.63	0.62	3.74	4.39	17.63	0.65	3.10
TPSSh	$\mu_{\mathrm{GS}}$	0.22	1.11	0.96	0.07	0.15	1.41	0.98	0.04	0.18	1.41	0.98	0.04
	$\mu_{\mathrm{ES}}$	1.21	4.70	0.95	0.39	3.96	15.04	0.52	3.25	3.04	15.04	0.67	2.28
	$\mu_{\mathrm{GS}}-\mu_{\mathrm{ES}}$	1.13	4.40	0.93	0.43	4.08	15.48	0.69	2.69	3.09	15.48	0.67	2.29
B3LYP	$\mu_{\mathrm{GS}}$	0.23	0.97	0.97	0.05	0.10	1.39	0.98	0.03	0.15	1.39	0.98	0.03
	$\mu_{\mathrm{ES}}$	0.75	2.78	0.96	0.20	2.65	10.96	0.65	1.96	2.01	10.96	0.75	1.36
	$\mu_{\mathrm{GS}}-\mu_{\mathrm{ES}}$	0.79	2.51	0.94	0.25	2.69	11.26	0.78	1.55	2.04	11.29	0.75	1.38
X3LYP	$\mu_{\mathrm{GS}}$	0.24	0.95	0.98	0.04	0.10	1.38	0.98	0.03	0.15	1.38	0.98	0.03
	$\mu_{\mathrm{ES}}$	0.72	2.37	0.96	0.19	2.42	10.08	0.68	1.72	1.84	10.08	0.77	1.20
	$\mu_{\mathrm{GS}}-\mu_{\mathrm{ES}}$	0.78	2.31	0.94	0.25	2.44	10.39	0.80	1.35	1.88	10.39	0.77	1.22
APF-D	$\mu_{\mathrm{GS}}$	0.23	0.94	0.97	0.05	0.10	1.37	0.98	0.03	0.14	1.37	0.98	0.03
	$\mu_{\mathrm{ES}}$	0.68	2.15	0.97	0.16	2.21	9.64	0.69	1.55	1.69	9.64	0.79	1.06
	$\mu_{\mathrm{GS}}-\mu_{\mathrm{ES}}$	0.77	2.44	0.95	0.23	2.23	9.96	0.82	1.21	1.73	9.96	0.78	1.10
PBE0	$\mu_{\mathrm{GS}}$	0.23	0.91	0.97	0.05	0.10	1.36	0.98	0.03	0.15	1.36	0.98	0.03
	$\mu_{\mathrm{ES}}$	0.69	1.85	0.97	0.16	1.93	8.63	0.73	1.30	1.51	8.63	0.82	0.89
	$\mu_{\mathrm{GS}}-\mu_{\mathrm{ES}}$	0.80	2.75	0.95	0.23	1.94	8.93	0.84	1.00	1.55	8.93	0.80	0.93
M06	$\mu_{\mathrm{GS}}$	0.23	0.91	0.97	0.05	0.13	1.47	0.98	0.04	0.17	1.47	0.98	0.04
	$\mu_{\mathrm{ES}}$	0.97	3.00	0.96	0.24	1.60	6.39	0.76	1.00	1.38	6.39	0.83	0.75
	$\mu_{\mathrm{GS}}-\mu_{\mathrm{ES}}$	1.08	3.91	0.92	0.40	1.65	6.76	0.86	0.78	1.45	6.76	0.80	0.85
SOGGA11-X	$\mu_{\mathrm{GS}}$	0.28	0.73	0.98	0.05	0.14	1.36	0.97	0.04	0.19	1.36	0.98	0.04
	$\mu_{\mathrm{ES}}$	1.18	4.08	0.97	0.27	0.54	2.19	0.90	0.26	0.77	4.08	0.93	0.28
	$\mu_{\mathrm{GS}}-\mu_{\mathrm{ES}}$	1.26	4.81	0.95	0.39	0.51	2.41	0.94	0.38	0.77	4.81	0.91	0.36
BMK	$\mu_{\mathrm{GS}}$	0.31	0.68	0.99	0.03	0.20	1.29	0.97	0.04	0.24	1.29	0.98	0.04
	$\mu_{\mathrm{ES}}$	1.00	3.45	0.98	0.18	0.52	2.30	0.90	0.24	0.69	3.45	0.95	0.23
	$\mu_{\mathrm{GS}}-\mu_{\mathrm{ES}}$	1.04	4.13	0.98	0.21	0.48	2.34	0.95	0.16	0.68	4.13	0.93	0.27
MN15	$\mu_{\mathrm{GS}}$	0.17	0.43	0.99	0.02	0.15	1.42	0.97	0.04	0.16	1.42	0.98	0.03
	$\mu_{\mathrm{ES}}$	1.18	4.19	0.98	0.22	0.52	2.38	0.91	0.23	0.76	4.19	0.94	0.26
	$\mu_{\mathrm{GS}}-\mu_{\mathrm{ES}}$	1.21	4.62	0.97	0.29	0.51	2.18	0.95	0.15	0.76	4.62	0.93	0.30
M06-2X	$\mu_{\mathrm{GS}}$	0.19	0.36	0.99	0.02	0.18	1.34	0.97	0.05	0.18	1.34	0.98	0.03
	$\mu_{\mathrm{ES}}$	1.19	4.24	0.98	0.23	0.71	3.13	0.90	0.30	0.89	4.24	0.95	0.27
	$\mu_{\mathrm{GS}}-\mu_{\mathrm{ES}}$	1.19	4.52	0.99	0.17	0.74	2.93	0.96	0.19	0.91	4.52	0.96	0.26
M06-HF	$\mu_{\mathrm{GS}}$	0.43	1.02	0.95	0.11	0.59	1.21	0.95	0.14	0.53	1.21	0.96	0.12
	$\mu_{\mathrm{ES}}$	1.70	6.16	0.92	0.66	1.91	8.74	0.06	2.37	1.86	8.74	0.66	1.42
	$\mu_{\mathrm{GS}}-\mu_{\mathrm{ES}}$	1.50	5.98	0.97	0.40	2.07	9.43	0.53	1.81	1.90	9.43	0.74	1.31
LC-BLYP	$\mu_{\mathrm{GS}}$	0.31	0.61	0.98	0.05	0.46	1.13	0.96	0.10	0.41	1.13	0.97	0.08
	$\mu_{\mathrm{ES}}$	1.87	6.51	0.92	0.72	1.95	7.56	0.08	2.35	1.96	7.56	0.65	1.49
	$\mu_{\mathrm{GS}}-\mu_{\mathrm{ES}}$	1.76	6.47	0.98	0.36	2.08	8.12	0.56	1.73	2.00	8.12	0.74	1.34
CAM-B3LYP	$\mu_{\mathrm{GS}}$	0.22	0.39	0.99	0.02	0.23	1.29	0.97	0.05	0.22	1.29	0.98	0.04
	$\mu_{\mathrm{ES}}$	1.47	5.15	0.97	0.34	0.95	3.62	0.83	0.49	1.15	5.15	0.92	0.43
	$\mu_{\mathrm{GS}}-\mu_{\mathrm{ES}}$	1.47	5.49	0.98	0.29	1.00	3.88	0.94	0.30	1.18	5.49	0.94	0.39
ωB97X	$\mu_{\mathrm{GS}}$	0.15	0.36	0.99	0.02	0.26	1.29	0.97	0.06	0.22	1.29	0.98	0.04
	$\mu_{\mathrm{ES}}$	1.77	6.51	0.94	0.61	1.74	6.57	0.32	1.78	1.78	6.57	0.75	1.15
	$\mu_{\mathrm{GS}}-\mu_{\mathrm{ES}}$	1.74	6.47	0.98	0.35	1.80	6.88	0.73	1.16	1.81	6.88	0.83	0.99
ωB97X-D	$\mu_{\mathrm{GS}}$	0.16	0.33	0.99	0.02	0.20	1.31	0.97	0.05	0.18	1.31	0.98	0.03
	$\mu_{\mathrm{ES}}$	1.62	6.04	0.96	0.45	1.33	5.21	0.64	1.00	1.46	6.04	0.86	0.72
	$\mu_{\mathrm{GS}}-\mu_{\mathrm{ES}}$	1.63	6.25	0.98	0.33	1.36	5.41	0.88	0.59	1.48	6.25	0.91	0.60

## Data Availability

The data presented in this study are available in this article or the Supplementary Material.

## Supplementary Materials

Figure S1: Difluoroborane structures (BF${}_{2}$) studied herein, Figures S2 and S3: HPIP structures studied herein, Table S1: Results of CC2 simulations performed in the gas phase-BF${}_{2}$ set, Table S2: Results of CC2 simulations performed in the gas phase-HPIP set, Figure S4: Mean Signed Errors (eV) determined for BF${}_{2}$ dyes excitation energies, with respect to CC2, Figure S5: Mean Signed Errors (eV) determined for HPIP dyes excitation energies, with respect to CC2, Figure S6: Percentage signed errors established for BF${}_{2}$ dipole moments, with respect to CC2, Figure S7: Percentage signed errors established for HPIP dipole moments, with respect to CC2, Table S3: Electron density difference plots and related electron transition parameters of the 13-BF${}_{2}$ dye, Table S4: Electron density difference plots and related electron transition parameters of the 27-BF${}_{2}$ dye, Table S5: Electron density difference plots and related electron transition parameters of the 45-HPIP dye, Table S6: Electron density difference plots and related electron transition parameters of the 13-HPIP dye, Tables S7 and S8: Differences between respective EDD plots, Table S9: Λ diagnostic results of the BF${}_{2}$ dyes set, Table S10: Λ diagnostic results of the HPIP dyes set.
