# Supplementary material for: Benchmarking Density Functional Approximations for Excited-State Properties of Fluorescent Dyes
Source: Molecules. 2021 Dec 8;26(24):7434. doi: 10.3390/molecules26247434 (PMC8703901; doi:10.3390/molecules26247434)
Supplement: Supplementary file 1 [file molecules-26-07434-s001.zip › molecules-1456866-supplementary.pdf]

Supporting Information: Benchmarking Density Functional Approximations for Excited-State Properties of Fluorescent Dyes

Anna M. Grabarz*,† and Borys Ośmiałowski*,‡

†*Faculty of Chemistry, Wrocław University of Science and Technology, Wyb. Wyspiańskiego 27, PL–50370 Wrocław, Poland*

‡*Faculty of Chemistry, Nicolaus Copernicus University, Gagarina 7, PL-87100 Toruń, Poland*

E-mail: anna.grabarz@pwr.edu.pl; borys.osmialowski@umk.pl

S1 Examined structures

S1.1 Difluoroboranes

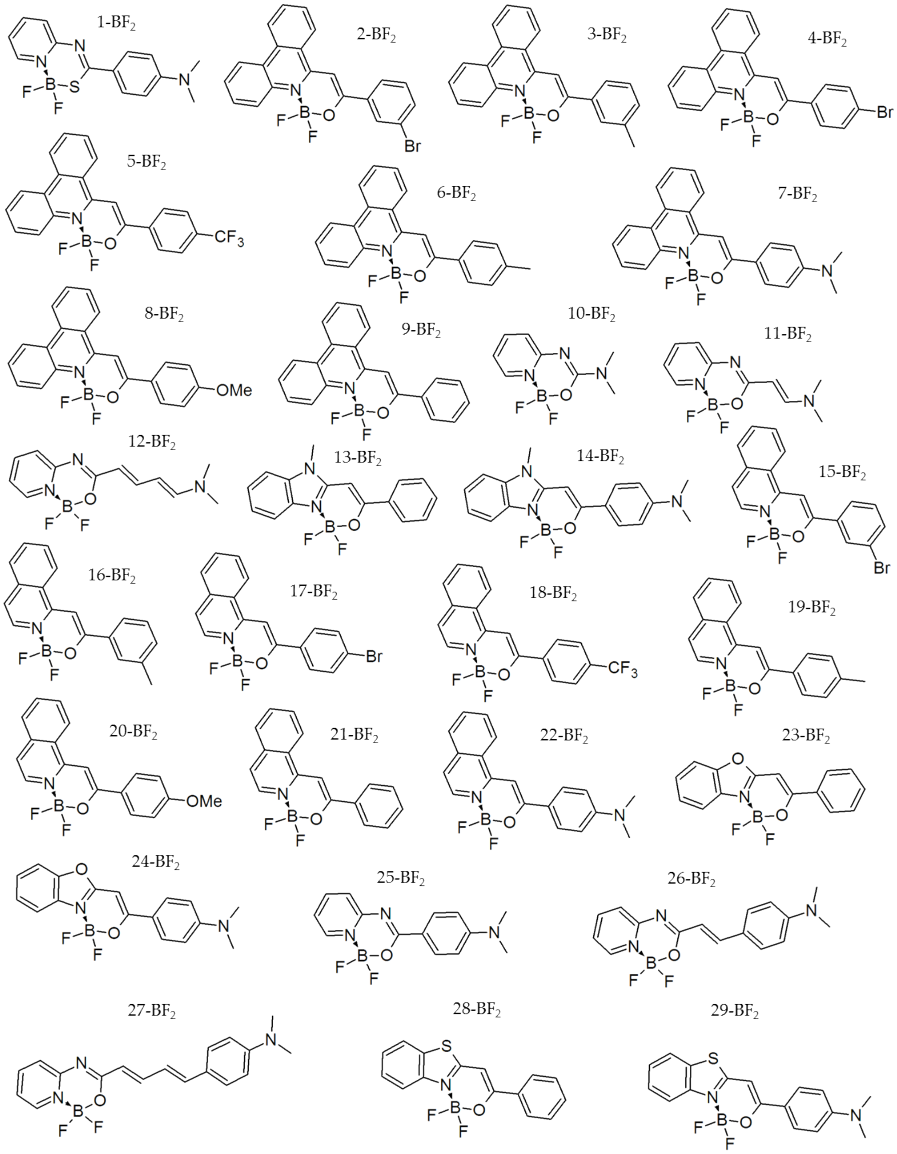

Figure S1: Difluoroborane structures (BF_2) studied herein.

S1.2 HPIP derivatives

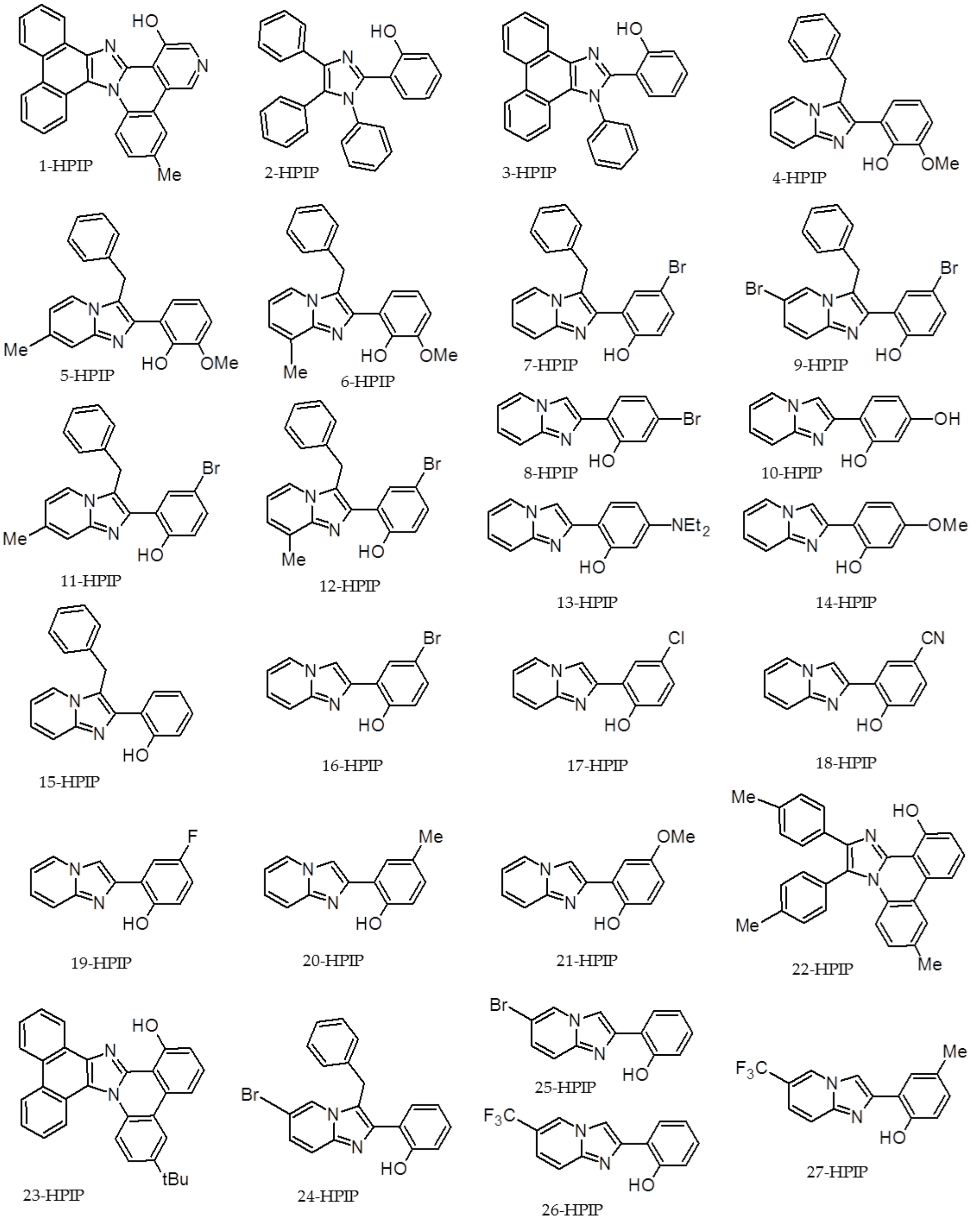

Figure S2: HPIP structures studied herein.

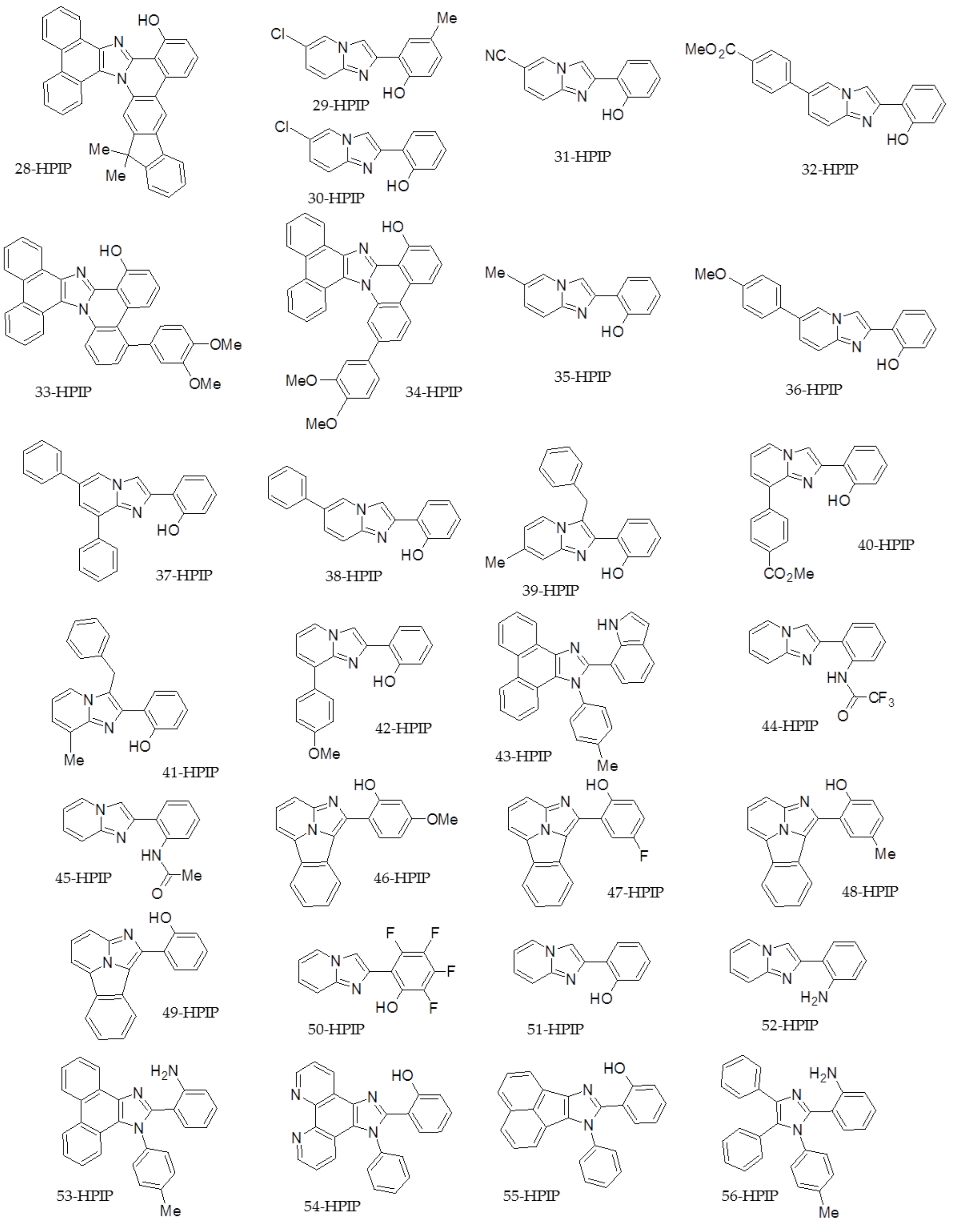

Figure S3: HPIP derivative structures studied herein

S2 Reference data

S2.1 Difluoroboranes

Table S1: CC2 simulations performed in the gas phase.

Struct.	Exc. energy	Dipole moments		
		μ_{GS}	μ_{ES}	μ_{ES}-μ_{GS}
1-BF_2	3.155	6.537	16.165	9.628
2-BF_2	3.216	7.525	6.021	-1.504
3-BF_2	3.199	5.445	4.107	-1.337
4-BF_2	3.196	6.443	4.822	-1.621
5-BF_2	3.223	7.352	6.267	-1.085
6-BF_2	3.188	5.823	4.656	-1.167
7-BF_2	2.940	6.991	11.764	4.773
8-BF_2	3.137	4.645	4.829	0.184
9-BF_2	3.216	5.863	4.412	-1.451
10-BF_2	4.002	4.325	5.703	1.378
11-BF_2	3.631	7.277	10.383	3.106
12-BF_2	3.324	8.850	15.121	6.271
13-BF_2	3.761	7.445	7.469	0.024
14-BF_2	3.420	8.281	11.831	3.550
15-BF_2	3.320	5.487	3.787	-1.700
16-BF_2	3.289	6.661	5.549	-1.112
17-BF_2	3.289	6.989	5.079	-1.911
18-BF_2	3.324	7.827	5.993	-1.834
19-BF_2	3.271	6.347	5.503	-0.844
20-BF_2	3.208	7.483	7.555	0.071
21-BF_2	3.303	6.413	5.105	-1.309
22-BF_2	2.999	7.151	12.113	4.962
23-BF_2	3.757	4.584	4.849	0.265
24-BF_2	3.367	6.417	11.748	5.331
25-BF_2	3.457	5.527	14.075	8.549
26-BF_2	3.204	7.371	17.489	10.118
27-BF_2	3.037	8.457	21.386	12.929
28-BF_2	3.523	4.547	4.466	-0.081
29-BF_2	3.179	6.344	11.534	5.190

S2.2 HPIP derivatives

Table S2: CC2 simulations performed in the gas phase.

Struct.	Exc. energy	Dipole moments		
		μ_{GS}	μ_{ES}	μ_{ES}-μ_{GS}
1-HPIP	3.632	5.696	6.515	0.819
2-HPIP	4.091	5.630	3.606	-2.024
3-HPIP	3.764	5.045	5.272	0.227
4-HPIP	3.815	4.079	5.489	1.410
5-HPIP	3.839	4.345	4.655	0.310
6-HPIP	3.897	3.683	5.118	1.434
7-HPIP	3.838	5.833	2.568	-3.265
8-HPIP	3.920	7.138	3.059	-4.079
9-HPIP	3.709	4.073	3.758	-0.315
10-HPIP	3.824	4.316	5.082	0.766
11-HPIP	3.855	6.401	2.491	-3.910
12-HPIP	3.906	5.669	2.115	-3.554
13-HPIP	3.516	3.196	12.782	9.586
14-HPIP	3.780	3.649	6.512	2.863
15-HPIP	3.846	5.252	4.454	-0.798
16-HPIP	3.897	5.864	1.560	-4.304
17-HPIP	3.898	5.826	1.484	-4.342
18-HPIP	4.002	7.780	2.528	-5.252
19-HPIP	3.878	5.569	1.594	-3.975
20-HPIP	3.845	5.120	4.820	-0.300
21-HPIP	3.708	4.193	4.545	0.351
22-HPIP	3.714	4.027	3.407	-0.620
23-HPIP	3.619	4.030	3.200	-0.830
24-HPIP	3.701	3.693	5.770	2.077
25-HPIP	3.750	3.486	5.961	2.475
26-HPIP	3.719	2.334	8.189	5.855
27-HPIP	3.785	2.260	7.028	4.768
28-HPIP	3.553	4.164	3.160	-1.004
29-HPIP	3.707	3.331	7.050	3.719
30-HPIP	3.769	3.410	5.884	2.473
31-HPIP	3.633	1.378	8.585	7.207
32-HPIP	3.703	5.117	7.536	2.419
33-HPIP	3.505	5.387	6.266	0.879
34-HPIP	3.554	4.683	3.766	-0.917
35-HPIP	3.919	5.868	3.894	-1.974
36-HPIP	3.798	7.030	4.727	-2.303
37-HPIP	3.645	5.023	6.128	1.105
38-HPIP	3.779	5.644	4.548	-1.097
39-HPIP	3.864	5.610	3.889	-1.721
40-HPIP	3.639	4.447	9.811	5.364
41-HPIP	3.931	4.915	3.990	-0.925
42-HPIP	3.798	3.442	5.810	2.368
43-HPIP	3.825	3.535	3.721	0.186
44-HPIP	4.100	7.401	6.115	-1.286
45-HPIP	4.016	5.520	5.630	0.110
46-HPIP	3.123	2.573	2.080	-0.493
47-HPIP	3.176	4.913	1.853	-3.059
48-HPIP	3.155	4.338	2.231	-2.107
49-HPIP	3.190	4.516	2.339	-2.177
50-HPIP	4.066	7.657	2.463	-5.194
51-HPIP	3.903	5.255	4.049	-1.206
52-HPIP	3.745	3.061	4.065	1.004
53-HPIP	3.763	3.178	5.638	2.460
54-HPIP	3.776	5.803	9.599	3.797
55-HPIP	2.945	5.179	7.031	1.853
56-HPIP	4.025	3.421	1.586	-1.835

S3 XCFs accuracy - vertical energy

	SVWN	BLYP	M06-L	TPSSh	B3LYP	X3LYP	APF-D	PBE0	M06	SOGGA11-X	BMK	MN15	M06-2X	M06-HF	LC-BLYP	CAM-B3LYP	ωB97X	ωB97X-D
	X=0	X=0	X=0	X=10	X=20	X=21.8	X=23	X=25	X=27	X=40.15	X=42	X=44	X=54	X=100	X=0-100	X=19-65	X=15.77-100	X=22.2-100
1-BF_2	0.728	0.691	0.458	0.302	0.139	0.101	0.063	0.019	0.005	-0.287	-0.225	-0.175	-0.300	-0.513	-0.607	-0.341	-0.534	-0.413
2-BF_2	0.459	0.449	0.254	0.205	0.132	0.109	0.081	0.054	0.085	-0.144	-0.132	-0.033	-0.149	-0.327	-0.408	-0.164	-0.306	-0.192
3-BF_2	0.427	0.423	0.231	0.190	0.120	0.098	0.070	0.043	0.073	-0.151	-0.138	-0.039	-0.152	-0.316	-0.405	-0.169	-0.308	-0.197
4-BF_2	0.459	0.453	0.262	0.215	0.141	0.118	0.090	0.063	0.093	-0.138	-0.124	-0.027	-0.143	-0.324	-0.404	-0.159	-0.303	-0.189
5-BF_2	0.457	0.456	0.269	0.224	0.148	0.125	0.098	0.071	0.102	-0.129	-0.119	-0.021	-0.141	-0.325	-0.399	-0.151	-0.295	-0.180
6-BF_2	0.425	0.420	0.230	0.189	0.120	0.098	0.071	0.044	0.072	-0.151	-0.136	-0.038	-0.150	-0.314	-0.403	-0.168	-0.307	-0.197
7-BF_2	0.589	0.547	0.349	0.244	0.123	0.093	0.068	0.034	0.025	-0.203	-0.171	-0.097	-0.204	-0.372	-0.490	-0.244	-0.403	-0.289
8-BF_2	0.466	0.452	0.256	0.201	0.120	0.096	0.069	0.041	0.061	-0.163	-0.144	-0.048	-0.160	-0.324	-0.416	-0.180	-0.322	-0.212
9-BF_2	0.420	0.419	0.229	0.189	0.120	0.098	0.071	0.044	0.073	-0.150	-0.137	-0.038	-0.150	-0.314	-0.402	-0.165	-0.304	-0.193
10-BF_2	0.624	0.577	0.288	0.194	0.090	0.059	0.017	-0.019	0.003	-0.272	-0.222	-0.107	-0.234	-0.363	-0.413	-0.223	-0.341	-0.248
11-BF_2	0.714	0.676	0.390	0.257	0.128	0.095	0.047	0.010	0.015	-0.249	-0.191	-0.093	-0.193	-0.266	-0.412	-0.219	-0.355	-0.262
12-BF_2	0.705	0.676	0.416	0.275	0.154	0.125	0.083	0.050	0.053	-0.181	-0.122	-0.046	-0.134	-0.237	-0.374	-0.169	-0.310	-0.214
13-BF_2	0.551	0.565	0.363	0.301	0.226	0.202	0.164	0.135	0.190	-0.074	-0.072	0.034	-0.086	-0.270	-0.341	-0.093	-0.245	-0.132
14-BF_2	0.520	0.495	0.268	0.215	0.133	0.110	0.073	0.044	0.074	-0.176	-0.149	-0.059	-0.164	-0.328	-0.432	-0.189	-0.349	-0.237
15-BF_2	0.432	0.431	0.244	0.209	0.149	0.128	0.102	0.078	0.122	-0.099	-0.092	0.008	-0.098	-0.254	-0.312	-0.105	-0.223	-0.125
16-BF_2	0.423	0.421	0.230	0.197	0.138	0.118	0.091	0.066	0.107	-0.110	-0.101	-0.002	-0.105	-0.251	-0.319	-0.116	-0.233	-0.137
17-BF_2	0.475	0.468	0.274	0.228	0.163	0.142	0.114	0.089	0.130	-0.094	-0.085	0.013	-0.095	-0.257	-0.316	-0.105	-0.227	-0.128
18-BF_2	0.450	0.450	0.265	0.231	0.166	0.145	0.119	0.094	0.137	-0.085	-0.079	0.020	-0.089	-0.252	-0.305	-0.094	-0.214	-0.115
19-BF_2	0.433	0.428	0.237	0.201	0.140	0.120	0.093	0.068	0.107	-0.110	-0.100	-0.001	-0.105	-0.252	-0.320	-0.116	-0.235	-0.139
20-BF_2	0.501	0.486	0.284	0.223	0.144	0.121	0.093	0.066	0.096	-0.126	-0.110	-0.014	-0.120	-0.271	-0.343	-0.135	-0.259	-0.162
21-BF_2	0.418	0.418	0.229	0.197	0.138	0.118	0.091	0.067	0.108	-0.108	-0.099	0.000	-0.103	-0.249	-0.316	-0.113	-0.229	-0.133
22-BF_2	0.643	0.600	0.396	0.280	0.155	0.125	0.098	0.064	0.061	-0.170	-0.141	-0.067	-0.170	-0.331	-0.430	-0.205	-0.350	-0.246
23-BF_2	0.523	0.535	0.333	0.279	0.205	0.182	0.145	0.116	0.157	-0.092	-0.079	0.029	-0.092	-0.233	-0.329	-0.100	-0.244	-0.136
24-BF_2	0.490	0.461	0.235	0.183	0.099	0.074	0.041	0.010	0.019	-0.216	-0.173	-0.087	-0.191	-0.328	-0.449	-0.218	-0.375	-0.266
25-BF_2	0.984	0.936	0.672	0.470	0.277	0.232	0.183	0.132	0.116	-0.212	-0.150	-0.081	-0.229	-0.463	-0.529	-0.255	-0.457	-0.330
26-BF_2	0.792	0.757	0.524	0.373	0.234	0.201	0.163	0.125	0.122	-0.140	-0.092	-0.029	-0.152	-0.386	-0.455	-0.182	-0.372	-0.248
27-BF_2	0.780	0.752	0.549	0.422	0.296	0.266	0.234	0.199	0.196	-0.050	-0.011	0.040	-0.082	-0.358	-0.410	-0.114	-0.313	-0.183
28-BF_2	0.387	0.401	0.204	0.175	0.121	0.100	0.067	0.042	0.085	-0.138	-0.121	-0.021	-0.124	-0.226	-0.341	-0.135	-0.258	-0.162
29-BF_2	0.410	0.383	0.177	0.131	0.050	0.026	-0.001	-0.030	-0.022	-0.237	-0.196	-0.117	-0.210	-0.320	-0.455	-0.241	-0.382	-0.281
MAE	0.541	0.525	0.314	0.241	0.151	0.125	0.093	0.063	0.085	-0.153	-0.128	-0.038	-0.149	-0.311	-0.398	-0.168	-0.312	-0.205

Figure S4: Errors (eV) determined for BF_2 dyes excitation energies, w.r.t CC2 benchmark. Calculations performed in gas phase. “X” under XCFs names denotes the fraction of Hartree-Fock exchange energy.

	SVWN	BLYP	M06-L	TPSSh	B3LYP	X3LYP	APF-D	PBE0	M06	SOGGA11-X	BMK	MN15	M06-2X	M06-HF	LC-BLYP	CAM-B3LYP	ωB97X	ωB97X-D
	X=0	X=0	X=0	X=10	X=20	X=21.8	X=23	X=25	X=27	X=40.15	X=42	X=44	X=54	X=100	X=0-100	X=19-65	X=15.77-100	X=22.2-100
1-HPIP	0.736	0.743	0.541	0.409	0.278	0.245	0.199	0.159	0.231	-0.099	-0.132	-0.026	-0.219	-0.610	-0.519	-0.193	-0.394	-0.245
2-HPIP	1.179	1.159	0.849	0.666	0.472	0.426	0.362	0.311	0.367	-0.024	-0.041	0.081	-0.123	-0.481	-0.408	-0.108	-0.291	-0.155
3-HPIP	0.914	0.891	0.616	0.430	0.277	0.241	0.196	0.156	0.223	-0.090	-0.109	-0.004	-0.164	-0.502	-0.437	-0.149	-0.321	-0.189
4-HPIP	1.279	1.248	0.992	0.715	0.456	0.398	0.340	0.277	0.324	-0.124	-0.118	-0.022	-0.210	-0.671	-0.605	-0.196	-0.431	-0.246
5-HPIP	1.227	1.196	0.928	0.662	0.410	0.356	0.299	0.240	0.302	-0.137	-0.129	-0.026	-0.211	-0.672	-0.612	-0.200	-0.433	-0.245
6-HPIP	1.227	1.205	0.932	0.685	0.425	0.370	0.316	0.254	0.299	-0.135	-0.131	-0.026	-0.216	-0.654	-0.596	-0.201	-0.429	-0.247
7-HPIP	1.067	1.042	0.772	0.539	0.360	0.315	0.257	0.208	0.270	-0.122	-0.116	-0.015	-0.190	-0.612	-0.557	-0.180	-0.396	-0.225
8-HPIP	0.966	0.967	0.694	0.519	0.371	0.331	0.273	0.227	0.293	-0.087	-0.092	0.012	-0.164	-0.576	-0.516	-0.145	-0.367	-0.196
9-HPIP	1.082	1.050	0.786	0.548	0.351	0.304	0.246	0.193	0.250	-0.161	-0.153	-0.051	-0.235	-0.683	-0.630	-0.232	-0.459	-0.281
10-HPIP	0.967	0.973	0.712	0.542	0.388	0.347	0.286	0.238	0.302	-0.097	-0.108	-0.006	-0.189	-0.651	-0.600	-0.180	-0.435	-0.247
11-HPIP	1.019	0.993	0.717	0.500	0.333	0.292	0.236	0.189	0.261	-0.128	-0.121	-0.016	-0.189	-0.608	-0.555	-0.181	-0.395	-0.223
12-HPIP	1.012	0.996	0.713	0.512	0.336	0.293	0.239	0.191	0.249	-0.132	-0.127	-0.019	-0.193	-0.583	-0.538	-0.183	-0.388	-0.225
13-HPIP	1.061	1.054	0.797	0.609	0.421	0.375	0.311	0.257	0.286	-0.135	-0.132	-0.070	-0.246	-0.688	-0.719	-0.275	-0.567	-0.375
14-HPIP	0.990	0.993	0.730	0.556	0.395	0.353	0.290	0.241	0.304	-0.105	-0.113	-0.016	-0.201	-0.675	-0.625	-0.194	-0.458	-0.266
15-HPIP	0.971	0.960	0.700	0.500	0.332	0.291	0.234	0.187	0.261	-0.125	-0.125	-0.024	-0.199	-0.643	-0.583	-0.185	-0.411	-0.230
16-HPIP	1.153	1.134	0.846	0.613	0.420	0.372	0.312	0.259	0.308	-0.092	-0.090	0.012	-0.163	-0.557	-0.502	-0.148	-0.359	-0.198
17-HPIP	1.146	1.125	0.835	0.615	0.419	0.372	0.313	0.260	0.315	-0.089	-0.089	0.011	-0.162	-0.560	-0.494	-0.145	-0.355	-0.194
18-HPIP	0.965	0.965	0.685	0.505	0.347	0.308	0.254	0.209	0.269	-0.092	-0.097	0.012	-0.158	-0.557	-0.494	-0.137	-0.346	-0.179
19-HPIP	1.152	1.138	0.866	0.639	0.426	0.378	0.324	0.271	0.315	-0.087	-0.087	0.013	-0.167	-0.572	-0.483	-0.144	-0.347	-0.192
20-HPIP	1.141	1.122	0.859	0.633	0.442	0.396	0.334	0.281	0.340	-0.079	-0.083	0.014	-0.170	-0.597	-0.525	-0.153	-0.384	-0.213
21-HPIP	1.355	1.324	1.061	0.791	0.524	0.464	0.408	0.341	0.368	-0.110	-0.097	-0.010	-0.209	-0.622	-0.518	-0.184	-0.386	-0.238
22-HPIP	0.824	0.817	0.592	0.425	0.268	0.229	0.176	0.130	0.182	-0.191	-0.200	-0.110	-0.297	-0.713	-0.657	-0.296	-0.515	-0.349
23-HPIP	0.756	0.751	0.549	0.407	0.271	0.238	0.191	0.151	0.226	-0.114	-0.139	-0.033	-0.218	-0.601	-0.531	-0.207	-0.408	-0.260
24-HPIP	0.965	0.947	0.693	0.490	0.307	0.264	0.208	0.159	0.229	-0.170	-0.168	-0.063	-0.246	-0.703	-0.649	-0.240	-0.472	-0.288
25-HPIP	1.046	1.033	0.762	0.563	0.372	0.327	0.267	0.216	0.277	-0.133	-0.139	-0.034	-0.219	-0.670	-0.624	-0.210	-0.456	-0.269
26-HPIP	1.097	1.078	0.801	0.600	0.407	0.362	0.300	0.247	0.304	-0.115	-0.110	-0.008	-0.197	-0.652	-0.603	-0.182	-0.434	-0.242
27-HPIP	1.181	1.149	0.889	0.661	0.459	0.411	0.347	0.291	0.345	-0.104	-0.094	0.001	-0.198	-0.659	-0.596	-0.186	-0.442	-0.255
28-HPIP	0.837	0.823	0.604	0.436	0.273	0.236	0.187	0.144	0.213	-0.144	-0.171	-0.066	-0.256	-0.645	-0.583	-0.249	-0.459	-0.307
29-HPIP	1.149	1.124	0.873	0.639	0.440	0.391	0.329	0.273	0.332	-0.109	-0.107	-0.014	-0.211	-0.675	-0.606	-0.201	-0.452	-0.269
30-HPIP	1.062	1.050	0.783	0.576	0.386	0.341	0.280	0.228	0.291	-0.121	-0.123	-0.023	-0.210	-0.663	-0.608	-0.197	-0.443	-0.257
31-HPIP	1.127	1.105	0.836	0.634	0.434	0.387	0.329	0.274	0.317	-0.094	-0.087	-0.002	-0.194	-0.684	-0.629	-0.178	-0.444	-0.241
32-HPIP	1.399	1.368	1.091	0.838	0.547	0.487	0.429	0.363	0.372	-0.070	-0.077	0.012	-0.199	-0.705	-0.663	-0.211	-0.487	-0.286
33-HPIP	0.802	0.759	0.538	0.396	0.245	0.209	0.166	0.124	0.174	-0.165	-0.188	-0.089	-0.276	-0.670	-0.612	-0.271	-0.484	-0.327
34-HPIP	0.859	0.818	0.597	0.452	0.295	0.258	0.213	0.170	0.231	-0.109	-0.141	-0.040	-0.234	-0.636	-0.550	-0.225	-0.437	-0.288
35-HPIP	1.045	1.039	0.758	0.563	0.386	0.344	0.281	0.232	0.306	-0.099	-0.111	-0.002	-0.184	-0.618	-0.558	-0.170	-0.403	-0.224
36-HPIP	1.014	0.995	0.727	0.538	0.360	0.319	0.262	0.214	0.280	-0.120	-0.127	-0.020	-0.207	-0.664	-0.609	-0.204	-0.449	-0.263
37-HPIP	1.073	1.042	0.794	0.593	0.383	0.336	0.288	0.235	0.270	-0.135	-0.144	-0.045	-0.246	-0.698	-0.695	-0.261	-0.530	-0.325
38-HPIP	1.060	1.039	0.772	0.569	0.377	0.334	0.278	0.228	0.286	-0.117	-0.124	-0.020	-0.207	-0.674	-0.623	-0.207	-0.456	-0.266
39-HPIP	0.936	0.923	0.657	0.471	0.314	0.276	0.219	0.175	0.259	-0.131	-0.129	-0.024	-0.198	-0.647	-0.592	-0.188	-0.416	-0.230
40-HPIP	1.318	1.287	1.035	0.812	0.550	0.494	0.443	0.379	0.384	-0.056	-0.069	0.012	-0.211	-0.682	-0.670	-0.227	-0.513	-0.305
41-HPIP	0.938	0.935	0.663	0.492	0.323	0.283	0.229	0.183	0.251	-0.129	-0.131	-0.024	-0.201	-0.624	-0.575	-0.187	-0.409	-0.229
42-HPIP	1.063	1.042	0.784	0.597	0.396	0.350	0.300	0.247	0.287	-0.112	-0.121	-0.022	-0.210	-0.554	-0.531	-0.206	-0.424	-0.258
43-HPIP	1.032	1.011	0.740	0.571	0.419	0.385	0.340	0.302	0.366	0.062	0.043	0.140	-0.021	-0.356	-0.307	-0.005	-0.188	-0.050
44-HPIP	0.876	0.878	0.605	0.457	0.329	0.295	0.245	0.205	0.274	-0.063	-0.078	0.026	-0.138	-0.300	-0.514	-0.119	-0.341	-0.154
45-HPIP	1.032	1.013	0.731	0.547	0.372	0.332	0.280	0.234	0.282	-0.071	-0.076	0.020	-0.143	-0.535	-0.533	-0.133	-0.361	-0.173
46-HPIP	0.558	0.543	0.339	0.242	0.141	0.114	0.082	0.052	0.097	-0.158	-0.149	-0.058	-0.168	-0.387	-0.368	-0.167	-0.289	-0.200
47-HPIP	0.724	0.695	0.477	0.335	0.187	0.155	0.126	0.090	0.127	-0.140	-0.133	-0.042	-0.161	-0.394	-0.365	-0.152	-0.277	-0.182
48-HPIP	0.717	0.681	0.470	0.325	0.190	0.159	0.126	0.090	0.133	-0.141	-0.132	-0.043	-0.161	-0.389	-0.364	-0.155	-0.281	-0.188
49-HPIP	0.621	0.599	0.378	0.263	0.148	0.120	0.088	0.056	0.106	-0.149	-0.144	-0.051	-0.162	-0.374	-0.355	-0.154	-0.273	-0.182
50-HPIP	1.153	1.131	0.846	0.599	0.393	0.350	0.301	0.252	0.304	-0.067	-0.078	0.037	-0.145	-0.571	-0.514	-0.117	-0.340	-0.157
51-HPIP	1.054	1.048	0.770	0.573	0.395	0.352	0.291	0.242	0.306	-0.088	-0.097	0.006	-0.172	-0.602	-0.545	-0.153	-0.386	-0.206
52-HPIP	1.144	1.125	0.846	0.658	0.462	0.415	0.356	0.303	0.336	-0.067	-0.058	0.020	-0.144	-0.525	-0.524	-0.148	-0.377	-0.203
53-HPIP	1.008	0.962	0.672	0.470	0.284	0.246	0.199	0.158	0.216	-0.106	-0.119	-0.019	-0.175	-0.507	-0.475	-0.168	-0.349	-0.207
54-HPIP	1.054	1.021	0.779	0.566	0.370	0.327	0.281	0.232	0.282	-0.068	-0.100	0.002	-0.185	-0.561	-0.476	-0.169	-0.356	-0.213
55-HPIP	0.871	0.854	0.676	0.542	0.419	0.389	0.358	0.324	0.355	0.093	0.080	0.142	-0.010	-0.441	-0.407	-0.019	-0.241	-0.060
56-HPIP	1.198	1.148	0.843	0.639	0.428	0.383	0.325	0.275	0.338	-0.057	-0.063	0.053	-0.128	-0.428	-0.426	-0.120	-0.297	-0.153
MAE	1.021	1.002	0.743	0.549	0.366	0.324	0.271	0.222	0.276	-0.104	-0.109	-0.010	-0.188	-0.589	-0.544	-0.179	-0.397	-0.230

Figure S5: Errors (eV) established for HPIP excitation energies, w.r.t CC2 benchmark. Calculations performed in gas phase. "X" under XCFs names denotes the fraction of Hartree-Fock exchange energy.

S4 XCFs accuracy - dipole moments

	SVWN		BLYP		M06-L		TPSSh		B3LYP		X3LYP		APF-D		PBE0		M06	
	X=0		X=0		X=0		X=10		X=20		X=21.8		X=23		X=25		X=27	
	GS	ES	GS	ES	GS	ES	GS	ES	GS	ES	GS	ES	GS	ES	GS	ES	GS	ES
1 - BF_2	-6.4	-12.5	-4.1	-11.5	-4.6	-11.6	-3.3	-2.4	-2.1	3.5	-2.0	4.7	-2.5	5.3	-2.4	6.5	-1.2	9.6
2 - BF_2	1.3	25.7	2.1	23.2	2.9	9.6	-1.3	-2.8	-2.4	-7.5	-2.7	-8.6	-2.4	-8.8	-2.6	-9.6	-1.7	-12.8
3 - BF_2	1.7	23.7	1.6	25.3	2.9	21.7	-1.0	10.8	-3.4	2.5	-3.9	0.9	-2.9	1.4	-3.2	0.0	-3.4	-2.6
4 - BF_2	2.9	23.3	3.7	21.5	4.7	11.6	0.6	-0.7	-0.9	-6.1	-1.3	-7.5	-0.8	-7.9	-1.1	-9.0	-0.4	-14.4
5 - BF_2	1.4	-29.9	2.9	-30.1	4.5	-34.3	1.0	-28.4	-0.8	-25.9	-1.2	-25.8	-0.2	-23.4	-0.4	-22.9	-0.8	-30.1
6 - BF_2	-0.5	16.0	0.7	20.3	0.7	16.8	-1.6	10.2	-4.0	3.1	-4.4	1.9	-3.9	1.7	-4.2	0.6	-4.8	-0.8
7 - BF_2	-7.5	-41.6	-4.7	-38.0	-4.9	-39.1	-4.6	-21.0	-4.9	-5.5	-5.0	-3.0	-5.2	-3.1	-5.2	-0.6	-5.0	7.6
8 - BF_2	-3.3	-53.4	-2.6	-42.6	-3.5	-36.0	-4.2	-11.2	-6.2	0.8	-6.5	2.2	-6.1	1.7	-6.4	2.8	-7.4	7.9
9 - BF_2	0.7	22.4	1.6	22.0	2.1	14.2	-0.6	6.6	-3.1	-1.2	-3.5	-2.5	-2.8	-2.3	-3.1	-3.4	-3.4	-7.1
10 - BF_2	-1.0	-60.9	0.2	-53.1	0.1	-51.4	-0.8	-32.0	-2.6	-19.5	-2.9	-17.3	-2.3	-16.9	-2.4	-14.7	-3.8	-8.3
11 - BF_2	-4.1	-58.7	-2.2	-55.5	-2.6	-56.3	-2.5	-36.8	-2.9	-19.8	-3.0	-16.5	-3.0	-15.6	-3.1	-12.3	-3.2	-4.9
12 - BF_2	-8.8	-43.7	-6.3	-41.6	-6.6	-41.8	-6.0	-21.9	-5.6	-4.9	-5.6	-1.9	-5.8	-0.7	-5.7	1.9	-5.7	7.1
13 - BF_2	0.8	-29.3	2.1	-29.6	2.7	-33.1	1.0	-21.6	-0.3	-16.3	-0.6	-15.5	-0.3	-14.9	-0.4	-14.1	0.5	-12.6
14 - BF_2	-4.8	-25.0	-2.8	-17.3	-3.0	-13.2	-3.0	-0.7	-3.5	7.0	-3.6	8.3	-3.7	7.5	-3.7	8.6	-3.1	15.7
15 - BF_2	4.0	8.3	5.5	9.8	6.7	-0.1	4.8	-5.4	2.0	-10.9	1.6	-12.2	2.6	-11.7	2.3	-12.6	1.7	-20.5
16 - BF_2	0.7	5.4	2.5	8.6	2.5	7.3	0.9	4.7	-1.3	0.2	-1.7	-0.6	-1.2	-0.6	-1.5	-1.3	-2.4	-4.2
17 - BF_2	4.1	4.1	5.2	8.9	6.2	10.2	2.4	2.4	0.9	-3.7	0.5	-5.1	1.0	-5.5	0.7	-6.7	1.1	-11.9
18 - BF_2	2.7	-22.2	4.3	-21.4	5.7	-26.0	2.5	-24.5	0.6	-23.3	0.2	-23.7	1.1	-22.1	0.9	-22.1	0.4	-28.9
19 - BF_2	1.2	-4.5	2.6	0.7	3.1	1.6	0.7	3.1	-1.4	0.1	-1.7	-0.5	-1.1	-0.1	-1.4	-0.5	-2.1	-2.3
20 - BF_2	0.3	-34.9	1.9	-29.2	2.3	-24.3	0.1	-8.7	-1.7	-1.8	-2.1	-1.0	-1.5	-0.6	-1.7	0.2	-2.5	1.6
21 - BF_2	2.1	11.2	3.3	12.5	4.1	10.5	1.5	4.8	-0.7	-0.5	-1.1	-1.5	-0.4	-1.2	-0.6	-2.1	-1.1	-5.5
22 - BF_2	-5.9	-39.6	-3.3	-36.9	-3.0	-10.5	-3.2	-21.6	-3.6	-7.2	-3.7	-4.8	-3.7	-4.3	-3.7	-1.7	-3.9	5.7
23 - BF_2	0.3	-50.1	0.8	-53.5	1.0	-61.4	-0.3	-38.8	-2.1	-28.0	-2.4	-26.6	-1.9	-25.4	-2.2	-24.0	-2.2	-24.2
24 - BF_2	-10.4	-19.9	-8.1	-14.4	29.3	-11.4	-7.3	2.3	-7.0	10.8	-7.0	12.2	-7.4	11.6	-7.3	12.9	-6.5	19.7
25 - BF_2	-9.4	-46.1	-6.8	-45.5	-6.4	-47.7	-5.3	-33.4	-4.9	-19.7	-4.8	-16.9	-4.9	-15.2	-4.7	-12.1	-4.8	-6.7
26 - BF_2	-14.9	-41.5	-12.0	-40.2	-11.3	-37.8	-9.6	-16.8	-8.5	-2.2	-8.4	0.3	-8.3	1.3	-8.0	3.6	-8.2	9.2
27 - BF_2	-19.5	-25.5	-16.3	-24.7	-15.3	-20.7	-13.2	-5.4	-11.5	4.7	-11.2	6.4	-11.1	7.0	-10.7	8.6	-10.7	14.0
28 - BF_2	-0.7	-38.1	-0.1	-40.6	2.8	-42.3	0.5	-26.5	-3.3	-22.1	-3.7	-21.4	-2.1	-18.9	-2.3	-18.0	-3.6	-20.4
29 - BF_2	-11.3	-16.8	-9.0	-11.8	30.3	-11.6	-7.5	0.8	-7.8	9.8	-7.7	11.2	-7.6	10.4	-7.5	11.8	-7.1	18.8
**MIN**	**-19.5**	**-60.9**	**-16.3**	**-55.5**	**-15.3**	**-61.4**	**-13.2**	**-38.8**	**-11.5**	**-28.0**	**-11.2**	**-26.6**	**-11.1**	**-25.4**	**-10.7**	**-24.0**	**-10.7**	**-30.1**
**MAE**	**-2.9**	**-19.1**	**-1.3**	**-16.7**	**1.8**	**-17.5**	**-2.0**	**-10.9**	**-3.2**	**-6.3**	**-3.4**	**-5.7**	**-3.0**	**-5.2**	**-3.2**	**-4.5**	**-3.3**	**-3.5**
**MAX**	**4.1**	**25.7**	**5.5**	**25.3**	**30.3**	**21.7**	**4.8**	**10.8**	**2.0**	**10.8**	**1.6**	**12.2**	**2.6**	**11.6**	**2.3**	**12.9**	**1.7**	**19.7**

	SOGGA11-X		BMK		MN15		M06-2X		M06-HF		LC-BLYP		CAM-B3LYP		ωB97X		ωB97X-D	
	X=40.15		X=42		X=44		X=54		X=100		X=0-100		X=19-65		X=15,7-100		X=22.2-100	
	GS	ES	GS	ES	GS	ES	GS	ES	GS	ES	GS	ES	GS	ES	GS	ES	GS	ES
1 - BF_2	-2.1	16.5	-0.8	15.0	1.0	19.0	1.7	20.5	2.7	38.1	1.6	36.1	0.9	24.4	2.2	34.2	1.5	29.0
2 - BF_2	-5.1	-15.9	-5.9	-15.1	-4.0	-13.1	-4.8	-13.3	-13.5	-22.7	-8.1	-20.6	-4.8	-15.5	-4.3	-15.9	-4.0	-14.8
3 - BF_2	-5.8	-8.8	-6.5	-10.5	-4.7	-9.0	-5.0	-11.7	-12.0	-28.7	-10.1	-26.5	-6.4	-14.2	-5.7	-17.9	-4.8	-13.3
4 - BF_2	-3.3	-17.3	-4.4	-15.9	-2.4	-13.8	-3.3	-13.9	-12.0	-22.4	-6.8	-23.7	-3.6	-17.9	-3.0	-18.7	-2.6	-17.6
5 - BF_2	-1.9	-21.7	-3.7	-20.7	-1.5	-17.5	-2.2	-13.3	-10.5	-11.7	-4.9	-14.0	-2.3	-16.6	-0.6	-10.7	-0.6	-12.8
6 - BF_2	-6.7	-6.8	-7.3	-9.1	-5.1	-7.0	-5.5	-10.0	-11.2	-25.1	-10.3	-23.1	-6.7	-11.3	-6.2	-15.0	-5.5	-10.7
7 - BF_2	-5.8	14.1	-6.1	10.6	-3.4	16.6	-3.5	17.1	-5.0	20.7	-5.3	25.1	-4.1	21.6	-3.0	26.9	-3.3	25.5
8 - BF_2	-8.8	6.8	-8.4	3.6	-6.8	6.0	-6.6	4.1	-10.0	-3.5	-10.4	-1.2	-7.9	6.1	-7.4	4.3	-7.0	7.5
9 - BF_2	-5.5	-11.4	-6.3	-12.4	-4.1	-10.3	-4.6	-12.4	-10.8	-26.7	-9.2	-25.8	-5.7	-15.3	-5.1	-18.2	-4.5	-14.5
10 - BF_2	-3.9	-1.2	-4.7	-3.1	-2.7	1.8	-3.0	3.2	-6.9	7.6	-5.4	13.2	-3.6	5.7	-2.4	12.0	-2.6	7.0
11 - BF_2	-3.8	3.3	-4.3	1.4	-1.7	5.6	-2.3	7.6	-4.2	9.4	-2.6	13.5	-2.2	10.2	-1.1	14.0	-1.7	11.9
12 - BF_2	-5.6	12.9	-5.9	10.5	-3.0	13.7	-2.9	13.3	-2.4	11.8	-1.6	16.8	-3.1	16.5	-1.1	18.4	-2.6	18.2
13 - BF_2	-1.6	-10.9	-2.8	-10.0	-0.1	-6.6	-1.3	-4.7	-6.4	-3.9	-3.8	-5.2	-1.7	-5.8	-1.0	-3.2	-0.9	-4.3
14 - BF_2	-4.4	14.9	-4.8	10.1	-2.0	14.7	-2.5	13.2	-4.6	13.0	-3.8	15.5	-3.0	15.6	-1.8	17.6	-2.3	17.2
15 - BF_2	1.4	-19.2	-0.2	-17.5	1.7	-15.6	1.3	-13.9	-3.9	-18.4	-2.0	-24.9	0.4	-19.1	1.7	-18.8	1.8	-17.3
16 - BF_2	-3.3	-6.6	-4.3	-7.4	-2.1	-5.7	-2.5	-6.5	-8.1	-16.4	-6.1	-15.3	-3.3	-8.3	-2.5	-9.6	-2.3	-7.6
17 - BF_2	-1.1	-15.7	-2.4	-14.6	-0.6	-13.0	-1.4	-13.1	-9.7	-23.7	-4.4	-24.1	-1.5	-17.0	-0.9	-18.6	-0.7	-16.9
18 - BF_2	-0.5	-24.1	-2.4	-23.1	-0.4	-20.1	-1.0	-17.1	-8.9	-20.5	-3.5	-21.7	-1.1	-20.5	0.4	-16.9	0.4	-17.6
19 - BF_2	-3.1	-4.2	-4.2	-5.6	-2.3	-4.1	-2.5	-4.7	-8.5	-14.4	-6.2	-12.6	-3.4	-5.9	-2.5	-6.9	-2.2	-4.9
20 - BF_2	-3.4	2.5	-4.5	0.5	-2.3	2.5	-2.7	2.7	-9.0	-3.3	-6.8	-1.5	-3.7	2.5	-2.8	3.0	-2.5	4.0
21 - BF_2	-2.3	-8.3	-3.4	-8.8	-1.5	-7.5	-1.9	-8.0	-8.2	-19.0	-5.5	-18.0	-2.7	-10.5	-1.8	-11.9	-1.5	-9.6
22 - BF_2	-3.8	13.7	-4.5	10.5	-2.1	15.7	-2.1	17.3	-4.8	22.3	-3.6	26.8	-2.6	21.4	-1.2	27.8	-1.7	25.4
23 - BF_2	-3.7	-17.1	-4.2	-12.8	-2.2	-9.6	-2.5	-5.7	-6.1	-1.9	-5.2	-4.6	-3.4	-7.2	-2.7	-3.1	-2.6	-5.1
24 - BF_2	-7.3	21.0	-6.6	15.9	-3.8	21.0	-3.6	19.1	-2.0	21.0	-2.6	24.2	-4.0	22.4	-2.1	25.7	-3.4	24.4
25 - BF_2	-4.1	8.9	-4.2	7.1	-1.6	12.5	-1.6	16.0	-2.0	29.9	-0.7	34.2	-1.7	21.2	0.7	32.5	-0.7	26.9
26 - BF_2	-6.8	16.9	-6.4	13.9	-3.6	17.8	-2.6	18.3	-0.2	25.4	-0.1	28.7	-3.0	22.4	0.4	28.7	-1.8	26.3
27 - BF_2	-8.7	19.1	-8.0	16.1	-5.1	19.6	-3.3	19.8	1.1	27.6	0.5	30.5	-4.0	24.1	0.4	30.4	-2.4	28.2
28 - BF_2	-3.5	-13.8	-4.4	-11.9	-1.6	-8.5	-3.8	-7.7	-8.2	-8.3	-6.9	-9.4	-4.8	-9.7	-2.2	-5.1	-2.3	-6.4
29 - BF_2	-7.2	20.5	-6.8	16.0	-4.0	21.1	-4.2	19.5	-2.9	21.8	-4.1	25.0	-4.9	23.0	-2.2	26.9	-3.4	25.6
**MIN**	**-8.8**	**-24.1**	**-8.4**	**-23.1**	**-6.8**	**-20.1**	**-6.6**	**-17.1**	**-13.5**	**-28.7**	**-10.4**	**-26.5**	**-7.9**	**-20.5**	**-7.4**	**-18.8**	**-7.0**	**-17.6**
**MAE**	**-4.2**	**-1.1**	**-4.8**	**-2.3**	**-2.5**	**0.9**	**-2.8**	**1.2**	**-6.5**	**-0.8**	**-4.8**	**0.6**	**-3.4**	**1.5**	**-2.0**	**3.9**	**-2.3**	**3.6**
**MAX**	**1.4**	**21.0**	**-0.2**	**16.1**	**1.7**	**21.1**	**1.7**	**20.5**	**2.7**	**38.1**	**1.6**	**36.1**	**0.9**	**24.4**	**2.2**	**34.2**	**1.8**	**29.0**

Figure S6: Percentage signed errors established for difluoroborane dipole moments, w.r.t CC2 benchmark. Calculations performed in the gas phase. “X” under XCFs names denotes the fraction of Hartree-Fock exchange energy.

	SVWN		BLYP		M06L		TPSSh		B3LYP		X3LYP		APF-D		PBE0		M06		SOGGA11-X		BMK		MN15		M06-2X		M06-HF		LC-BLYP		CAM-B3LYP		wB97X		wB97X-D	
	X=0		X=0		X=0		X=10		X=20		X=21.8		X=23		X=25		X=27		X=40.15		X=42		X=44		X=54		X=100		X=0-100		X=19-65		X=15,7-100		X=22.2-100	
	GS	ES	GS	ES	GS	ES	GS	ES	GS	ES	GS	ES	GS	ES	GS	ES	GS	ES	GS	ES	GS	ES	GS	ES	GS	ES	GS	ES	GS	ES	GS	ES	GS	ES	GS	ES
1 -HPIP	0.1	-41.6	-3.4	-50.5	6.3	-52.0	2.9	-43.6	1.5	-39.4	1.3	-38.1	1.9	-33.2	1.9	-31.4	2.6	-32.2	2.2	-22.4	-1.3	-19.1	2.3	-14.1	1.4	-3.7	-5.9	12.5	-4.0	7.6	-0.5	-4.6	0.7	4.5	0.5	10.2
2 -HPIP	1.5	-64.7	-4.7	-75.8	8.1	-53.3	2.9	-34.7	2.1	7.8	1.8	18.6	1.7	33.3	1.6	45.4	4.5	57.4	1.7	60.7	-0.6	63.9	1.8	58.8	-0.2	34.4	-9.7	-30.5	-3.3	-26.5	-0.3	10.8	0.5	-1.0	0.2	-15.9
3 -HPIP	2.0	19.0	-5.0	-51.8	8.7	17.9	2.8	3.1	1.7	0.7	1.3	0.0	1.3	-3.8	1.1	-2.6	3.9	9.0	0.8	8.8	-1.4	6.6	1.0	11.9	-1.1	7.8	-12.1	2.1	-5.5	11.6	-1.5	11.7	-0.7	12.8	-1.3	14.1
4 -HPIP	4.8	-137.6	-6.1	-138.7	8.9	-141.5	1.0	-125.9	-2.4	-95.5	-3.1	-87.2	-2.4	-82.0	-2.9	-71.7	-0.8	-57.2	-5.8	-0.8	-8.8	-3.1	-6.4	4.0	-8.8	13.5	-24.1	20.8	-17.8	27.5	-9.7	16.7	-8.1	23.0	-10.4	27.3
5 -HPIP	3.0	-171.9	-4.9	-174.5	6.2	-174.6	0.5	-151.9	-2.9	-101.2	-3.6	-88.7	-3.2	-82.0	-3.6	-67.8	-2.1	-49.5	-6.4	7.2	-9.1	5.0	-6.8	10.2	-8.8	16.8	-21.2	17.7	-17.6	26.7	-9.9	19.4	-8.6	23.7	-10.9	27.7
6 -HPIP	6.4	-153.9	-6.3	-155.0	10.3	-156.6	0.8	-136.0	-2.4	-96.7	-3.1	-87.0	-2.1	-80.9	-2.6	-69.5	0.4	-54.4	-5.3	2.3	-8.0	0.4	-6.5	6.2	-8.8	17.3	-25.2	31.0	-18.0	40.3	-9.8	21.6	-7.9	29.1	-10.4	37.1
7 -HPIP	2.0	-245.5	-3.8	-247.5	4.5	-235.9	0.6	-166.8	-0.2	-94.0	-0.4	-78.6	-0.7	-63.4	-1.0	-48.0	0.2	-32.8	-2.6	-2.7	-4.0	-3.9	-2.6	0.9	-4.0	-7.4	-11.6	-69.0	-8.6	-60.1	-4.2	-14.1	-4.1	-25.8	-5.4	-38.8
8 -HPIP	3.1	-117.2	-4.4	-122.6	5.2	-112.1	0.4	-68.9	-0.1	-37.7	-0.4	-32.0	-0.7	-22.0	-1.0	-16.6	0.6	-13.6	-3.1	-7.0	-4.3	-7.9	-3.1	-4.9	-4.5	-11.4	-13.7	-82.9	-8.9	-76.6	-4.5	-17.2	-4.6	-28.8	-5.7	-46.7
9 -HPIP	3.5	-197.5	-5.8	-199.1	6.9	-195.9	4.8	-160.9	2.0	-111.8	1.6	-100.4	1.9	-90.3	1.6	-77.2	1.6	-60.2	0.8	5.2	-1.8	3.2	0.4	9.0	-1.6	27.1	-6.2	49.0	-8.6	47.9	-3.2	34.0	-2.5	44.2	-4.6	49.8
10 -HPIP	9.1	-58.4	-9.9	-62.0	12.9	-62.9	5.0	-48.5	1.2	-32.0	0.4	-28.8	1.6	-24.4	1.1	-20.6	1.4	-17.4	-2.9	9.1	-5.9	12.8	-3.6	16.1	-6.0	25.7	-21.7	56.2	-16.5	58.5	-7.7	32.4	-6.4	45.5	-9.3	54.7
11 -HPIP	-0.3	-222.5	-2.4	-226.9	1.7	-201.8	-0.2	-118.9	-1.2	-34.6	-1.4	-21.0	-1.9	-8.5	-2.1	1.5	-1.3	7.6	-3.7	-16.6	-5.0	-15.7	-3.2	-13.3	-4.4	-29.6	-10.0	-106.6	-8.8	-103.5	-4.8	-38.9	-4.8	-54.3	-5.7	-74.2
12 -HPIP	1.6	-301.3	-3.0	-304.5	3.7	-281.2	0.0	-187.4	-0.5	-92.4	-0.7	-74.4	-1.0	-56.9	-1.2	-40.1	0.4	-23.6	-2.5	-11.3	-3.7	-10.7	-2.4	-8.6	-3.8	-23.7	-11.1	-113.6	-7.7	-107.8	-4.0	-34.3	-3.9	-51.0	-4.8	-75.6
13 -HPIP	4.4	-18.3	-6.2	-18.8	10.6	-20.7	2.8	-17.1	-0.3	-10.9	-1.0	-9.6	0.0	-8.2	-0.4	-6.4	2.1	-2.1	-3.5	9.6	-6.1	9.3	-4.1	16.0	-5.9	21.4	-21.6	68.4	-17.5	59.2	-8.2	28.4	-6.5	40.7	-9.5	51.4
14 -HPIP	9.2	-44.0	-9.8	-46.4	12.5	-47.6	4.1	-37.2	-0.2	-24.7	-1.1	-22.2	-0.2	-18.4	-0.8	-15.2	0.6	-13.1	-4.5	10.2	-7.0	12.2	-6.1	16.5	-8.6	24.9	-23.9	60.1	-19.3	61.4	-10.0	31.1	-8.9	44.4	-12.2	54.2
15 -HPIP	3.4	-88.4	-5.3	-90.4	7.0	-85.8	2.7	-60.5	0.9	-28.8	0.5	-23.7	0.6	-18.9	0.3	-13.8	1.9	-8.7	-1.3	3.9	-3.4	3.2	-1.2	6.7	-3.1	7.4	-11.9	0.4	-9.0	5.4	-3.8	7.1	-3.2	7.7	-4.7	8.3
16 -HPIP	2.6	-509.8	-4.2	-518.5	4.6	-501.6	1.1	-397.9	0.3	-280.4	-0.1	-251.8	-0.2	-217.5	-0.5	-185.0	0.4	-156.5	-2.2	-14.3	-3.4	-16.7	-2.5	-5.4	-3.6	-12.7	-10.6	-180.1	-8.4	-171.3	-4.0	-27.6	-3.9	-56.2	-5.3	-110.1
17 -HPIP	2.9	-498.9	-4.4	-507.8	3.9	-486.4	1.5	-398.4	0.3	-276.9	0.0	-248.9	-0.1	-216.7	-0.3	-184.7	0.1	-160.7	-1.9	-16.5	-4.0	-10.4	-2.5	-2.8	-3.2	-14.0	-9.0	-182.1	-8.6	-185.9	-4.1	-30.1	-4.0	-61.2	-5.5	-120.2
18 -HPIP	-2.1	-115.2	0.6	-124.4	-1.0	-101.1	-1.6	-17.5	-2.3	61.2	-2.5	65.8	-2.5	62.8	-2.6	54.5	-3.1	38.1	-3.6	-24.2	-4.8	-28.7	-2.9	-24.3	-2.9	-42.7	-5.2	-133.7	-6.7	-153.2	-4.4	-56.8	-4.4	-78.0	-4.9	-109.9
19 -HPIP	3.3	-408.4	-4.9	-422.0	5.8	-419.2	2.1	-350.8	0.0	-244.7	-0.4	-221.4	-0.1	-200.2	-0.4	-173.7	-0.1	-143.9	-2.3	-17.4	-4.0	-12.1	-2.6	-3.5	-3.8	-0.6	-11.4	-153.8	-10.2	-160.1	-5.0	-13.2	-4.2	-38.3	-5.9	-94.3
20 -HPIP	4.1	-115.0	-5.7	-115.9	7.4	-117.4	2.9	-97.3	0.9	-72.3	0.4	-66.4	0.7	-59.4	0.4	-52.2	1.7	-47.8	-1.5	-4.5	-3.4	-3.7	-1.5	3.4	-3.2	14.9	-12.1	23.9	-9.8	25.6	-4.3	19.0	-3.6	27.0	-5.4	31.7
21 -HPIP	3.8	-196.3	-5.5	-198.4	7.0	-200.7	2.7	-188.0	0.7	-164.7	0.2	-158.0	0.5	-149.0	0.1	-139.4	1.5	-132.4	-1.9	-46.0	-3.2	-45.0	-2.1	-30.0	-3.6	2.3	-11.5	38.8	-10.0	41.1	-4.6	14.8	-4.1	31.3	-6.1	42.7
22 -HPIP	-0.6	-30.8	-4.3	-33.8	5.5	-44.6	2.6	-33.2	1.3	-26.2	1.0	-24.6	0.6	-22.9	0.4	-20.9	2.4	-18.6	-0.1	-7.4	-2.4	-3.6	1.1	3.8	-1.1	10.0	-9.7	20.2	-5.0	27.7	-1.3	18.0	-0.7	23.8	-1.0	29.3
23 -HPIP	0.7	-39.9	-3.7	-66.3	5.0	-91.9	0.9	-70.7	-1.3	-58.7	-1.6	-56.1	-1.6	-47.4	-1.8	-43.7	-0.2	-43.1	-3.9	-22.5	-6.4	-13.7	-0.7	-9.6	-4.1	4.6	-16.0	-9.9	-11.5	-0.9	-5.7	4.2	-4.9	9.3	-5.4	7.7
24 -HPIP	5.2	-83.1	-7.9	-84.8	10.9	-83.5	7.3	-68.3	3.4	-40.6	2.9	-35.6	3.8	-31.8	3.4	-26.3	4.2	-18.8	2.6	4.6	-1.0	4.6	2.4	7.0	-0.1	13.9	-8.1	24.5	-8.5	29.8	-2.1	18.1	-0.6	24.3	-2.8	28.3
25 -HPIP	6.3	-84.5	-8.4	-87.0	11.4	-86.8	8.5	-72.9	3.6	-47.9	3.0	-43.1	4.3	-38.3	3.9	-32.7	4.4	-27.0	3.0	3.8	-0.3	4.7	2.8	7.8	0.5	17.8	-5.6	41.1	-9.9	49.7	-3.0	24.4	-1.1	35.4	-3.9	44.2
26 -HPIP	6.0	-64.0	-9.1	-64.3	12.8	-66.5	6.5	-58.1	3.6	-46.1	3.2	-43.2	2.8	-38.7	2.5	-34.7	8.2	-34.1	0.2	-1.4	-1.6	-2.8	2.6	2.6	-0.6	14.4	-12.2	50.2	-11.7	56.6	-3.3	20.9	-3.4	33.4	-6.1	46.6
27 -HPIP	10.4	-73.7	-11.5	-75.2	16.4	-74.7	8.5	-62.7	5.6	-43.6	5.2	-39.7	4.8	-34.2	4.3	-29.4	12.1	-27.6	2.3	3.5	1.0	2.1	3.5	6.1	0.5	15.3	-10.7	42.5	-12.2	51.1	-2.9	21.1	-3.3	32.8	-7.0	43.0
28 -HPIP	1.3	-108.8	-4.3	-129.1	6.2	-142.9	2.0	-95.3	0.0	-66.8	-0.3	-62.6	0.0	-53.0	-0.1	-48.2	1.0	-53.3	-1.5	-27.2	-4.7	-10.2	0.4	-6.6	-2.7	9.0	-14.0	-14.5	-9.2	-2.2	-3.9	6.7	-2.9	10.2	-3.5	7.6
29 -HPIP	5.5	-73.1	-8.2	-74.0	14.0	-78.2	7.0	-67.1	3.0	-52.1	2.4	-48.5	3.3	-44.5	2.9	-39.8	4.9	-36.8	1.0	-2.5	0.8	-3.5	2.6	2.7	-2.0	16.5	-14.6	51.1	-10.4	55.9	-3.5	22.9	-1.6	35.4	-4.0	47.5
30 -HPIP	5.6	-85.3	-8.0	-87.9	14.1	-89.5	7.3	-73.5	3.2	-49.5	2.7	-44.7	3.6	-39.6	3.2	-33.9	5.4	-29.1	1.6	3.1	1.7	2.1	2.8	6.7	-1.5	17.7	-13.1	42.6	-9.8	48.7	-3.1	23.3	-1.3	34.6	-3.7	43.3
31 -HPIP	-22.5	-71.4	19.3	-72.5	-14.8	-73.5	-16.0	-63.7	-13.7	-47.7	-13.4	-44.2	-12.1	-41.1	-11.3	-37.0	-9.1	-33.0	-10.9	-9.4	-14.0	-11.1	-3.2	-5.3	-3.3	3.2	-22.0	28.9	-5.5	30.5	-4.6	6.6	-1.7	16.0	2.0	24.1
32 -HPIP	8.5	-228.6	-11.1	-229.8	12.7	-225.3	8.6	-199.6	6.5	-145.4	6.1	-133.8	6.2	-127.9	5.9	-114.5	7.3	-84.8	4.4	-29.1	1.3	-27.7	3.4	-16.8	1.2	5.7	-8.3	41.8	-6.9	48.6	-0.2	16.8	0.0	31.6	-2.3	41.8
33 -HPIP	-0.6	-462.8	-2.5	-301.4	5.0	-158.3	1.2	-80.8	0.8	-76.1	0.6	-73.2	0.2	-63.9	0.0	-59.4	2.8	-56.3	0.0	-30.5	-1.8	-21.1	1.6	-14.0	-0.4	8.0	-9.4	14.2	-5.6	17.7	-1.8	13.6	-1.3	26.5	-1.8	26.5
34 -HPIP	-2.5	-412.4	-1.1	-279.3	3.0	-39.5	2.2	-71.2	3.2	-60.6	3.3	-57.8	2.9	-55.0	3.0	-51.5	5.0	-43.3	4.3	-29.3	2.5	-23.1	5.6	-17.6	4.4	-6.2	1.3	0.0	2.8	-8.9	3.9	-7.2	4.3	-1.0	4.6	-2.9
35 -HPIP	1.4	-121.9	-4.1	-126.5	4.5	-119.8	2.4	-92.7	0.4	-56.1	0.0	-49.5	0.0	-40.1	-0.3	-32.8	0.8	-28.9	-1.8	3.5	-3.5	4.6	-1.1	7.9	-2.8	14.0	-9.4	4.9	-8.5	-0.7	-3.9	14.8	-3.5	17.2	-4.7	14.3
36 -HPIP	0.9	-110.0	-3.2	-110.6	4.3	-100.5	2.4	-71.3	1.7	-36.0	1.6	-30.6	1.4	-24.7	1.3	-19.2	2.5	-14.2	1.0	6.4	-0.6	5.5	1.5	9.1	0.3	12.6	-4.1	5.8	-3.7	-3.3	-0.8	13.0	-0.6	14.8	-1.3	12.2
37 -HPIP	3.4	-86.0	-4.6	-86.4	6.5	-85.0	2.1	-67.6	-0.1	-43.9	-0.5	-39.4	-0.2	-36.5	-0.5	-31.5	1.2	-24.2	-1.9	2.2	-3.9	2.2	-2.1	6.1	-4.1	17.7	-12.6	47.0	-11.1	35.8	-5.6	24.5	-4.9	34.6	-6.6	42.9
38 -HPIP	3.9	-159.8	-5.5	-160.6	7.3	-154.2	2.9	-114.8	0.8	-66.2	0.4	-58.0	0.7	-51.8	0.4	-43.1	1.4	-33.4	-1.1	7.1	-3.2	7.3	-1.4	11.8	-3.1	22.3	-11.5	33.4	-9.5	31.1	-4.4	28.6	-3.8	36.5	-5.3	39.7
39 -HPIP	1.6	-94.5	-4.2	-98.5	4.7	-86.3	2.0	-55.1	0.1	-17.0	-0.3	-12.2	-0.4	-7.5	-0.6	-3.3	0.5	0.1	-2.2	4.6	-4.1	3.5	-1.8	6.4	-3.5	4.5	-10.2	-11.2	-9.4	-8.2	-4.4	2.8	-3.9	0.7	-5.3	-0.7
40 -HPIP	-1.6	-86.0	1.5	-85.9	1.9	-83.7	-4.4	-76.4	-6.8	-60.7	-7.3	-57.4	-6.4	-55.3	-6.7	-51.3	-3.9	-42.2	-9.0	-20.0	-10.0	-19.6	-7.2	-13.8	-8.8	1.7	-18.9	38.7	-15.0	42.4	-10.4	9.2	-8.8	22.1	-9.6	34.0
41 -HPIP	4.1	-102.5	-5.2	-104.9	7.5	-96.6	2.5	-66.8	0.9	-30.6	0.6	-25.2	0.9	-19.8	0.6	-14.4	2.8	-8.7	-0.7	4.7	-2.6	4.3	-0.9	6.5	-2.7	8.7	-11.7	2.4	-8.4	6.3	-3.5	8.3	-2.7	9.0	-4.2	10.3
42 -HPIP	4.6	-88.5	-4.5	-88.6	6.6	-86.3	1.7	-71.0	-1.5	-48.3	-2.1	-44.1	-1.3	-40.4	-1.8	-35.5	0.7	-29.5	-4.1	0.8	-5.9	-0.6	-4.8	3.5	-7.1	20.2	-18.7	68.2	-16.1	62.8	-8.7	31.8	-7.5	44.6	-10.1	66.6
43 -HPIP	-3.3	-157.2	-2.3	-146.0	4.8	-173.8	0.9	-118.8	-0.1	-43.6	-0.4	-32.1	-1.4	-27.1	-1.6	-16.9	1.5	14.2	-0.8	18.8	-3.2	15.8	0.5	22.5	-2.5	16.7	-12.5	14.2	-7.6	19.5	-3.6	21.2	-3.6	21.6	-4.1	21.8
44 -HPIP	-2.0	-38.5	-0.2	-38.8	2.2	-25.7	-1.0	-0.8	-2.1	1.4	-2.4	0.7	-1.7	2.4	-1.8	1.7	-1.2	-0.9	-2.3	-4.4	-4.5	-5.3	-2.0	-2.8	-3.0	-2.8	-9.6	-6.2	-6.5	-9.8	-4.0	-5.7	-2.9	-5.2	-3.1	-5.3
45 -HPIP	-0.6	-97.4	-0.8	-99.1	4.6	-91.4	-0.4	-60.7	-1.8	-29.1	-2.1	-24.2	-1.4	-18.6	-1.5	-13.9	0.7	-9.6	-2.2	1.2	-4.0	1.9	-1.7	5.0	-2.1	7.0	-9.4	12.9	-7.5	6.1	-4.3	4.4	-3.5	7.2	-4.0	7.9
46 -HPIP	15.6	-182.7	-16.0	-178.6	21.3	-168.4	7.4	-107.4	0.2	-50.9	-1.1	-42.1	0.6	-40.3	-0.4	-31.8	1.3	-17.2	-5.4	14.6	-9.8	12.4	-8.9	19.7	-13.1	24.6	-40.0	26.5	-30.3	27.3	-15.4	27.9	-13.8	32.7	-18.7	33.6
47 -HPIP	4.4	-157.7	-6.3	-160.4	8.2	-154.0	3.1	-71.4	0.4	45.3	-0.1	63.3	0.5	62.9	0.2	75.3	0.8	57.7	-1.6	-13.6	-4.1	-7.4	-2.4	-11.6	-4.2	-31.7	-15.1	-89.9	-12.2	-82.5	-5.6	-44.3	-4.5	-55.7	-6.5	-66.1
48 -HPIP	6.0	-193.2	-7.9	-189.5	10.6	-187.9	4.2	-126.5	1.3	-58.7	0.7	-47.8	1.3	-45.1	0.9	-34.8	2.0	-24.4	-1.2	-14.4	-3.9	-15.6	-2.0	-11.3	-4.1	-18.3	-17.2	-53.1	-13.5	-49.0	-5.7	-25.8	-4.8	-30.8	-7.1	-36.7
49 -HPIP	5.1	-124.9	-7.1	-121.7	9.7	-102.6	4.1	-33.7	1.3	19.3	0.8	20.6	1.3	22.2	1.0	21.3	2.3	12.5	-0.8	-12.1	-3.4	-11.9	-1.5	-10.5	-3.5	-18.2	-15.4	-48.3	-12.3	-45.7	-5.1	-26.4	-4.1	-31.1	-6.2	-35.2
50 -HPIP	1.9	-179.0	-3.6	-184.4	4.5	-182.7	0.1	-89.9	-2.5	10.1	-3.1	18.6	-2.1	19.4	-2.4	22.4	-3.7	18.5	-4.7	-27.7	-7.3	-34.1	-5.4	-31.2	-5.9	-39.9	-15.8	-116.1	-12.3	-111.6	-7.0	-54.5	-5.5	-64.8	-6.6	-78.1
51 -HPIP	3.7	-120.4	-5.4	-124.0	6.9	-119.9	2.9	-93.0	0.9	-57.5	0.5	-50.8	0.8	-42.7	0.5	-35.3	1.8	-30.0	-1.3	3.0	-3.1	3.9	-1.4	8.2	-2.9	14.6	-11.0	15.0	-9.4	16.7	-4.0	16.3	-3.4	21.6	-5.1	23.1
52 -HPIP	9.1	-143.0	-10.1	-149.3	10.5	-149.1	5.9	-131.6	3.1	-103.9	2.5	-97.4	2.7	-87.3	2.2	-78.7	2.9	-72.1	-1.2	-7.9	-3.0	-6.0	-1.9	3.7	-3.5	27.2	-13.8	78.7	-13.1	79.7	-5.3	41.1	-5.3	65.7	-8.4	93.2
53 -HPIP	0.4	-96.7	-5.5	-74.6	7.1	-103.2	3.0	-60.3	1.9	-47.6	1.5	-44.2	0.5	-43.0	0.2	-34.0	2.9	-10.2	-1.4	9.1	-2.5	9.8	1.6	20.7	-0.7	18.0	-11.5	31.9	-5.5	36.1	-1.4	28.6	-1.7	31.7	-2.2	35.7
54 -HPIP	-0.8	-71.8	-2.0	-67.6	5.2	-65.5	0.1	-49.1	-1.0	-30.4	-1.3	-27.9	-1.1	-28.0	-1.3	-25.3	0.4	-19.1	-2.0	-8.0	-3.7	-5.0	-0.9	-0.4	-2.0	9.4	-10.9	40.4	-7.0	34.3	-3.5	16.0	-2.7	21.3	-2.9	31.0
55 -HPIP	1.7	-58.4	-4.4	-51.8	8.3	-44.6	2.8	-29.4	1.7	-15.9	1.5	-14.2	1.6	-14.1	1.5	-12.4	4.3	-6.4	1.6	-3.4	-1.0	-4.4	2.2	-0.1	-0.2	0.3	-10.4	6.5	-3.9	11.4	-0.7	3.7	0.3	7.2	0.1	10.4
56 -HPIP	1.5	-726.2	-6.3	-693.6	9.0	-681.2	4.4	-552.5	3.2	-347.9	2.9	-310.3	2.4	-301.0	2.1	-260.6	5.1	-200.8	1.4	-19.1	-1.0	-32.4	3.1	-16.3	0.6	18.8	-10.5	-47.0	-3.9	-31.1	0.0	15.6	0.1	0.4	-0.4	-16.2
MIN	-22.5	-726.2	-16.0	-693.6	-14.8	-681.2	-16.0	-552.5	-13.7	-347.9	-13.4	-310.3	-12.1	-301.0	-11.3	-260.6	-9.1	-200.8	-10.9	-46.0	-14.0	-45.0	-8.9	-31.2	-13.1	-42.7	-40.0	-182.1	-30.3	-185.9	-15.4	-56.8	-13.8	-78.0	-18.7	-120.2
MAE	2.7	-155.4	-4.9	-153.2	7.1	-141.9	2.4	-106.5	0.4	-64.3	0.0	-56.5	0.2	-49.9	-0.1	-41.8	1.6	-33.4	-1.6	-4.7	-3.7	-4.2	-1.2	0.9	-3.2	5.8	-13.1	-5.1	-9.9	-2.9	-4.4	6.3	-3.6	8.1	-5.1	6.2
MAX	15.6	19.0	19.3	-18.8	21.3	17.9	8.6	3.1	6.5	61.2	6.1	65.8	6.2	62.9	5.9	75.3	12.1	57.7	4.4	60.7	2.5	63.9	5.6	58.8	4.4	34.4	1.3	78.7	2.8	79.7	3.9	41.1	4.3	65.7	4.6	93.2

Figure S7: Percentage signed errors established for HPIP dipole moments, w.r.t CC2 benchmark. Calculations performed in gas phase. "X" under XCFs names denotes the fraction of Hartree-Fock exchange energy.

S5 EDD plots and charge transfer diagnostic

Table S3: Electron density difference plots and related electron transition parameters of 13-BF_2 dye (See ESI **Fig S1**). Presented results were obtained with aug-cc-pVDZ basis set in vacuum. Red (blue) color marks electron density depletion (gain) upon photon absorption. The contour value was set to 0.002 au.

DFA	EDD plots	d_{CT}	q_{CT}	$\mu_{ES} - \mu_{GS}$
		[Å]	[e]	[D]
BLYP	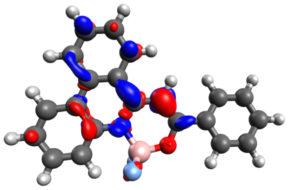	1.233	0.394	-2.328
PBE0	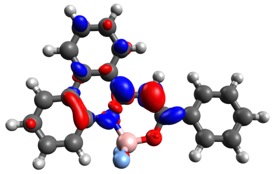	0.742	0.420	-1.480
MN15	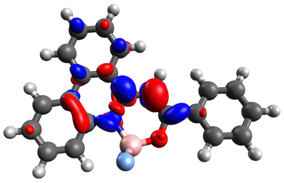	0.588	0.437	-1.234
ωB97X-D	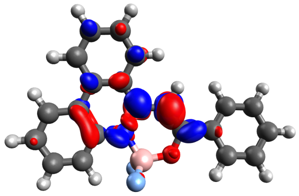	0.485	0.461	-1.072

Table S4: Electron density difference plots and related electron transition parameters of 27-BF_2 dye (See ESI **Fig S1**). Presented results were obtained with aug-cc-pVDZ basis set in vacuum. Red (blue) color marks electron density depletion (gain) upon photon absorption. The contour value was set to 0.002 au.

DFA	EDD plots	d_{CT} [Å]	q_{CT} [e]	$\mu_{ES} - \mu_{GS}$ [D]
BLYP	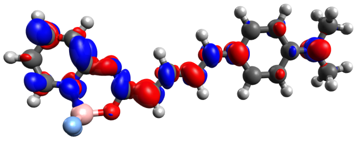	4.991	0.768	16.833
PBE0	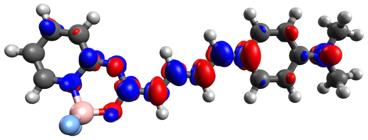	3.982	0.587	10.175
MN15	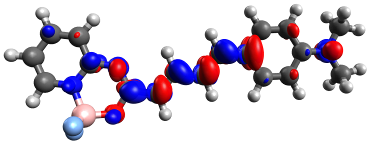	3.508	0.552	8.306
ωB97X-D	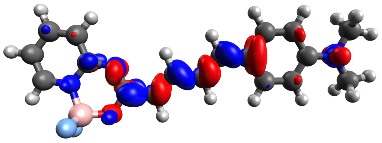	2.970	0.531	6.456

Table S5: Electron density difference plots and related electron transition parameters of 45-HPIP dye (See ESI **Fig S3**). Presented results were obtained with aug-cc-pVDZ basis set in vacuum. Red (blue) color marks electron density depletion (gain) upon photon absorption. The contour value was set to 0.003 au.

DFA	EDD plots	d_{CT} [Å]	q_{CT} [e]	$\mu_{ES} - \mu_{GS}$ [D]
BLYP	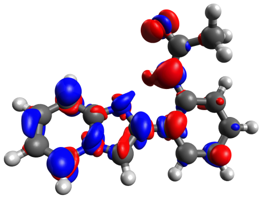	3.069	0.898	5.735
PBE0	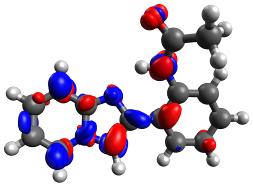	2.313	0.631	0.814
MN15	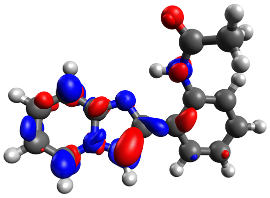	1.723	0.560	-0.268
ωB97X-D	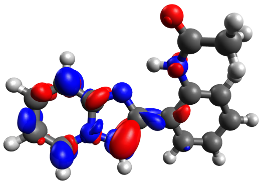	1.416	0.550	-0.558

Table S6: Electron density difference plots and related electron transition parameters of 13-HPIP dye (See ESI **Fig S2**). Presented results were obtained with aug-cc-pVDZ basis set in vacuum. Red (blue) color marks electron density depletion (gain) upon photon absorption. The contour value was set to 0.003 au.

DFA	EDD plots	d_{CT} [Å]	q_{CT} [e]	$\mu_{ES} - \mu_{GS}$ [D]
BLYP	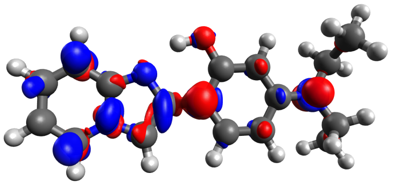	3.901	0.811	12.183
PBE0	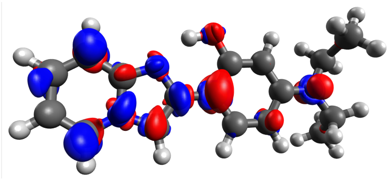	3.597	0.811	10.395
MN15	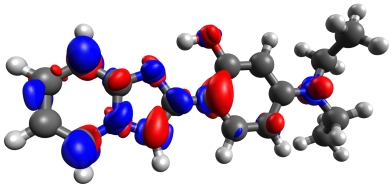	3.195	0.741	7.408
ωB97X-D	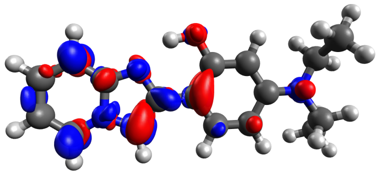	2.575	0.651	4.173

S5.1 Differences between respective EDD plots

Table S7: Differences between respective EDD plots. The contour values were set to 0.002 au and 0.003 au for BF_2 and HPIP dyes respectively.

Struct.	Diffrence EDD plots			ES
	$\Delta\rho$(MN15)-$\Delta\rho$BLYP	$\Delta\rho$(MN15)-$\Delta\rho$(PBE0)	$\Delta\rho$(MN15)-$\Delta\rho$(ωB97X-D)	
13-BF_2	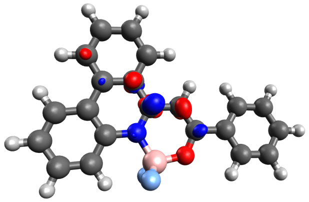	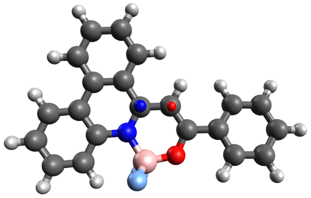	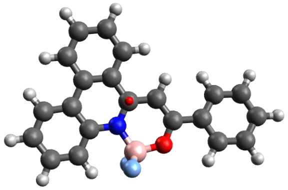	LC
27-BF_2	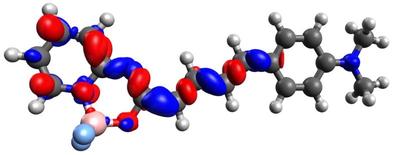	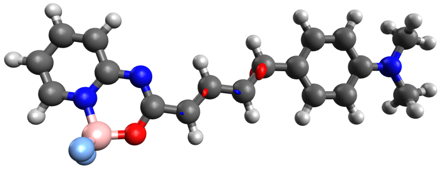	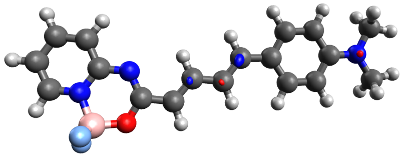	CT
45-HPIP	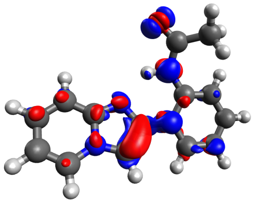	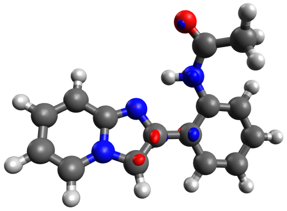	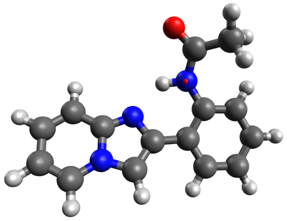	LC
13-HPIP	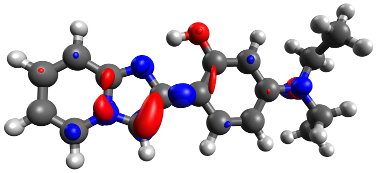	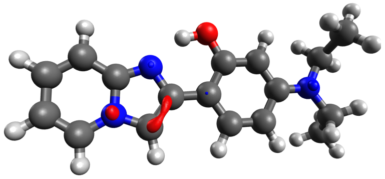	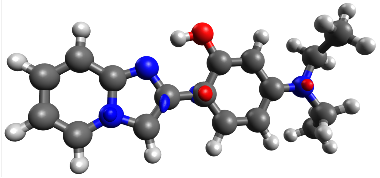	CT

Table S8: Differences between respective EDD plots. Note that the contour value was lowered to 0.001 au.

Struct.	Diffrence EDD plots			ES
	$\Delta\rho$(MN15)-$\Delta\rho$BLYP	$\Delta\rho$(MN15)-$\Delta\rho$(PBE0)	$\Delta\rho$(MN15)-$\Delta\rho$(ωB97X-D)	
13-BF_2	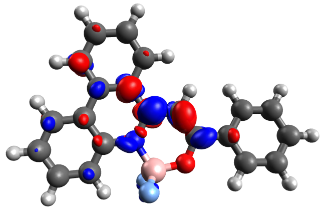	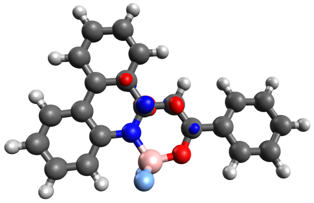	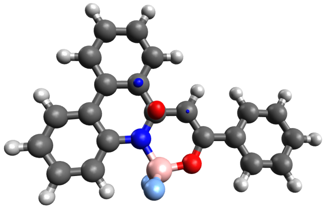	LC
27-BF_2	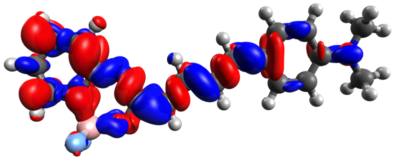	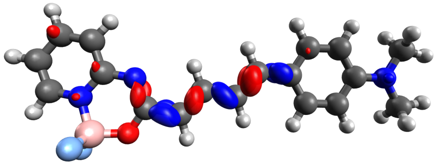	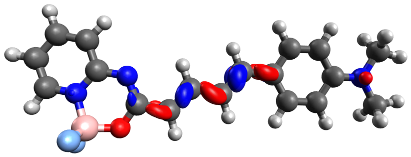	CT
45-HPIP	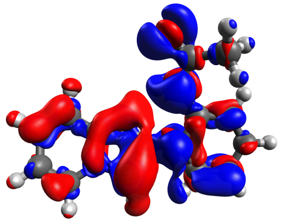	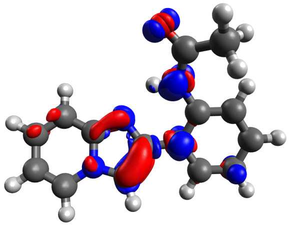	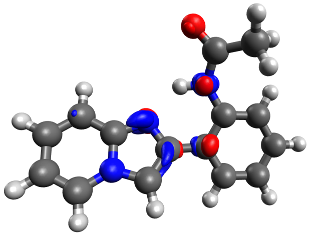	LC
13-HPIP	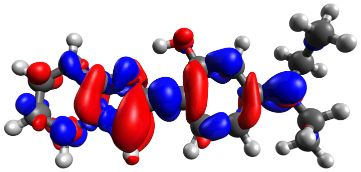	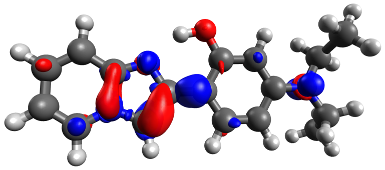	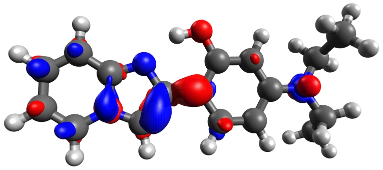	CT

S6 Λ diagnostic

Λ quantity represents the degree of spatial overlap between the occupied and virtual orbitals involved in an excitation, which allows to distinguish between local, charge-transfer and Rydberg excitations. By definition, Λ values fall between 0 and 1 and a small value of Λ indicates a long-range excitation; on the other hand a large value signifies a short-range (local) excitation.

Table S9: Λ diagnostic results of BF_2 dyes set. All results were calculated at CAM-B3LYP/aug-cc-pVDZ theory level.

Structure	Λ	Structure	Λ
1-BF2	0.528	16-BF2	0.72
2-BF2	0.703	17-BF2	0.726
3-BF2	0.696	18-BF2	0.738
4-BF2	0.702	19-BF2	0.715
5-BF2	0.707	20-BF2	0.686
6-BF2	0.694	21-BF2	0.724
7-BF2	0.596	22-BF2	0.597
8-BF2	0.674	23-BF2	0.711
9-BF2	0.7	24-BF2	0.629
10-BF2	0.648	25-BF2	0.528
11-BF2	0.589	26-BF2	0.583
12-BF2	0.621	27-BF2	0.614
13-BF2	0.718	28-BF2	0.724
14-BF2	0.662	29-BF2	0.635
15-BF2	0.729		

Table S10: Λ diagnostic results of HPIP dyes set. All results were calculated at CAM-B3LYP/aug-cc-pVDZ theory level.

Structure	Λ	Structure	Λ
1-HPIP	0.664	29-HPIP	0.499
2-HPIP	0.598	30-HPIP	0.513
3-HPIP	0.717	31-HPIP	0.515
4-HPIP	0.487	32-HPIP	0.433
5-HPIP	0.510	33-HPIP	0.602
6-HPIP	0.501	34-HPIP	0.607
7-HPIP	0.543	35-HPIP	0.528
8-HPIP	0.564	36-HPIP	0.532
9-HPIP	0.517	37-HPIP	0.486
10-HPIP	0.522	38-HPIP	0.528
11-HPIP	0.572	39-HPIP	0.568
12-HPIP	0.564	40-HPIP	0.433
13-HPIP	0.465	41-HPIP	0.563
14-HPIP	0.512	42-HPIP	0.524
15-HPIP	0.542	43-HPIP	0.716
16-HPIP	0.537	44-HPIP	0.657
17-HPIP	0.543	45-HPIP	0.599
18-HPIP	0.590	46-HPIP	0.642
19-HPIP	0.547	47-HPIP	0.644
20-HPIP	0.523	48-HPIP	0.630
21-HPIP	0.490	49-HPIP	0.643
22-HPIP	0.541	50-HPIP	0.597
23-HPIP	0.676	51-HPIP	0.540
24-HPIP	0.518	52-HPIP	0.507
25-HPIP	0.512	53-HPIP	0.668
26-HPIP	0.518	54-HPIP	0.663
27-HPIP	0.502	55-HPIP	0.594
28-HPIP	0.689	56-HPIP	0.542
